# Design, Synthesis, and Characterization of Novel Cannabidiol-Based Derivatives with Potent Antioxidant Activities

**DOI:** 10.3390/ijms25179579

**Published:** 2024-09-04

**Authors:** Eliav Peretz, Sanaa Musa

**Affiliations:** 1 Department of Biotechnology, Tel-Hai Academic College, Kiryat Shmona 11016, Israel; 2 Natural Compounds and Organic Synthesis Laboratory, Migal-Galilee Research Institute, Kiryat Shmona 11016, Israel

**Keywords:** cannabidiol, Schiff base, semicarbazones, antioxidant activities, low-density lipoprotein oxidation

## Abstract

In recent years, extensive research has focused on cannabidiol (CBD), a well-studied non-psychoactive component of the plant-derived cannabinoids. CBD has shown significant therapeutic potential for treating various diseases and disorders, including antioxidants and anti-inflammatory effects. Due to the promising therapeutic effect of CBD in a wide variety of diseases, synthetic derivatization of this compound has attracted the attention of drug discovery in both industry and academia. In the current research, we focused on the derivatization of CBD by introducing Schiff base moieties, particularly (thio)-semicarbazide and aminoguanidine motifs, at the 3-position of the olivetolic ring. We have designed, synthesized, and characterized new derivatives based on CBD’s framework, specifically aminoguanylhydrazone- and (thio)-semicarbazones-CBD-aldehyde compounds. Their antioxidant potential was assessed using FRAP and DPPH assays, alongside an evaluation of their effect on LDL oxidation induced by Cu^2+^ and AAPH. Our findings suggest that incorporating the thiosemicarbazide motif into the CBD framework produces a potent antioxidant, warranting further investigation.

## 1. Introduction

**Figure 1:**
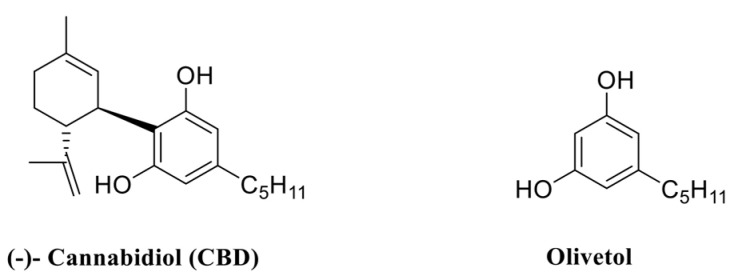
Chemical structure of (-)-cannabidiol.

Natural product-based medicine plays a pivotal role in medicinal aspects, emphasizing exploring natural components found in medicinal plants as a fundamental basis for creating innovative bioactive compounds [1]. One of the most interesting natural compounds is cannabidiol (CBD). CBD, a non-psychotropic, second major component of plant-derived phytocannabinoids, is undoubtedly the most interesting for its involvement in diverse biological activities, with antioxidant, anticancer, anti-inflammatory, anticonvulsant, and immunomodulatory effects [2,3,4,5,6]. The antioxidant capacity of CBD depends on the presence of the resorcinol group, which is responsible for transferring hydrogen atoms or electrons to the oxidants, and on the limonene ring and the n-pentyl moiety that contribute to the stability of the cation free radical or its semiquinone forms (Figure 1) [7].

The antioxidant properties of the phytocannabinoids, particularly CBD, have been extensively studied. CBD has been shown to reduce oxidative stress by scavenging free radicals and modulating the activity of antioxidant enzymes [8]. Additionally, it protects against oxidative damage in various cell types, including neurons, which is beneficial for neurodegenerative disorders like Alzheimer’s and Parkinson’s diseases [9,10,11,12]. The antioxidant properties of the phytocannabinoids and their ability to prevent low-density lipoprotein (LDL) oxidation have also been previously reported [13]. Studies have indicated that CBD can prevent the formation of conjugated dienes during LDL oxidation by extending the lag time of the initial stages, albeit with moderate activity [14]. LDL is widely recognized as a major cholesterol carrier in the bloodstream, and the concentration of LDL-cholesterol is directly correlated with the incidence of cardiovascular diseases [15,16]. Studies have extensively shown that oxidized LDL (ox-LDL) contributes significantly to the formation and progression of atherosclerotic plaques through various pathways. These pathways involve the activation and dysfunction of endothelial cells, along with the development of macrophage foam cells, which play a pivotal role in the initiation and development of early stages of atherosclerosis [17]. Recent research also suggests that oxLDL may exhibit anti-inflammatory and pro-inflammatory properties depending on the duration of oxLDL exposure and the degree of oxidation [18]. 

Due to the promising therapeutic effects of CBD in a wide variety of diseases, synthetic derivatization of this compound has attracted the attention of drug discovery in both industry and academia, with the aim of improving the potency, efficacy, or pharmacokinetic properties of this interesting phytocannabinoid [19,20,21]. Synthetic cannabinoids are used in pharmacological studies involving structure-activity relationships (SARs), receptor binding, and detailed mechanisms of these drugs [22,23]. However, most studies conducted to date have focused on the pentyl side chain of the olivetolic ring in CBD rather than on other positions of the ring (Figure 1) [24,25]. Only recent studies have focused on the chemical modification at the 3-position of CBD [26,27,28,29]. 

**Figure 2:**
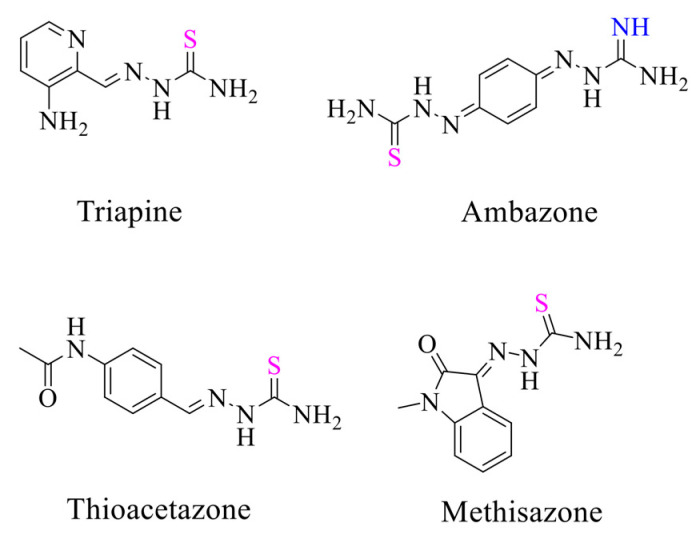
Compounds incorporated Schiff base motifs.

**Figure 3:**
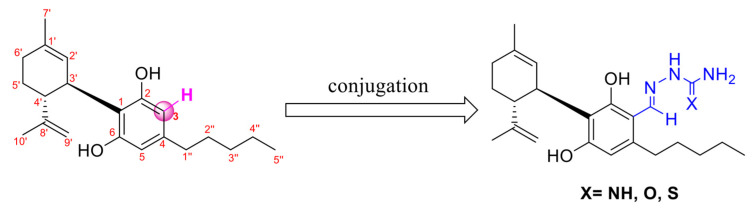
General structure of the Schiff base–cannabidiol conjugate.

Recently, research focusing on Schiff bases, particularly aminoguanylhydrazone and (thio)-semicarbazone motifs, has become an area of increasing interest, with a broad spectrum of biological activities, including antibacterial, antifungal, antiviral, antimalarial, anticancer, and antioxidant activities [30,31,32,33]. Intensive studies have revealed that incorporating a (thio)-semicarbazide moiety into another bioactive compound significantly enhances its biological activity compared to the parent compound. The first clinically approved drug of this class of compounds was p-acetamidobenzaldehyde thiosemicarbazone (Thioacetazone, Figure 2), synthesized by Behnisch et al. [34], which is still used for the treatment of multidrug-resistant tuberculosis [35]. N-methylisatin thiosemicarbazone (Methiazone, Figure 2) and 3-aminopyridine-2-carboxaldehyde thiosemicarbazone (Triapine, Figure 2) are two other clinically investigated compounds. Methiazone was developed against the smallpox virus but is no longer administered due to the development of a vaccine [36]. On the other hand, triapine, which is now in clinical trials for cancer treatment, has the potential to stop the growth of cancer cells by blocking an enzyme needed for cell growth [37]. Another interesting compound is 1,4-benzoquinone guanylhydrazone thiosemicarbazone (Ambazone, Figure 2), which contains two types of Schiff base, namely aminoguanylhydrazone and thiosemicarbazone. It has antiseptic properties, as reflected by its increased activity against gram-positive cocci bacteria. In addition, ambazone has been discovered to exhibit activity against various transplantable tumors in mice and rats [38]. As a result, medicinal chemists are showing a growing interest in semicarbazide-based scaffolds.

Currently, there is considerable interest in combining two or more pharmacophores from specific ligands to generate multiple ligands. These pharmacophores can be linked using a cleavable or non-cleavable linker or, more commonly, by overlapping them by taking advantage of structural commonalities [39]. Considering that both CBD and Schiff base pharmacophores are promising candidates for generating attractive molecular and structural libraries of bioactive compounds, we herein are highly interested in conjugating aminoguanidine/(thio)-semicarbazide moieties at the 3-position of the olivetolic ring of CBD to establish a unique chemical structure with superior oxidation inhibitors (Figure 3). 

To the best of our knowledge, no systematic study has described the chemical modification with Schiff base at the 3-position of CBD and assessed their antioxidant activities. Therefore, as part of our ongoing research, we designed, synthesized, and characterized aminoguanylhydrazone and (thio)-semicarbazone derivatives based on the cannabidiol framework. These derivatives exhibited strong antioxidant properties, potentially contributing to improved health by protecting against and inhibiting oxidative damage.

## 2. Results

### 2.1. Synthesis

**Scheme 1:**
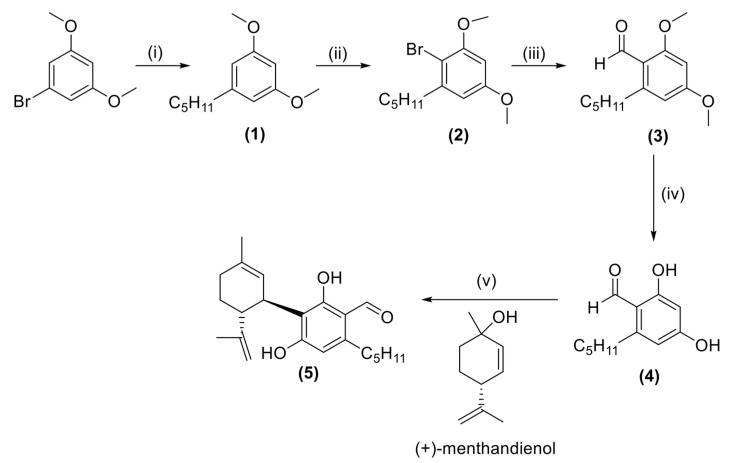
Synthesis pathway of the preparation of 3-CBD-aldehyde (**5**). Reagents and conditions: (i) (a) Mg_(s)_, THF, 40 °C, 1 h (b) bromopentane, 65 °C, 1 h. (ii) *N*-bromosuccinimide, dichloromethane, 30 min, RT. (iii) (a) Mg_(s)_, THF, 40 °C, 1 h (b) *N*,*N*-dimethylformamide, 65 °C, 1 h. (iv) AlCl_3_, dichloromethane, 24 h, RT. (v) *p*-toluene sulfonic acid, dichloromethane, 5 °C, 1 h.

The 3-formyl cannabidiol (CBD-aldehyde, **5**) was synthesized through a multi-step process. Initially, the commercially available 1-bromo-3,5-dimethoxybenzene was reacted in the presence of magnesium to form a Grignard reagent, followed by a transition metal cross-coupling reaction using primary alkyl halide, utilizing copper and lithium salts as pre-catalysts, resulting in 1,3-dimethoxy-5-pentylbenzene (**1**). Subsequently, a mono-bromination reaction with *N*-bromosuccinimde was then performed to obtain 2-bromo-1,5-dimethoxy-3-pentylbenzene (**2**) with a quantitative yield. The selectivity of the bromination reaction was confirmed through ^1^H-NMR analysis, wherein the two methoxy groups exhibited different chemical shifts (3.81 ppm vs. 3.76 ppm). Another Grignard reaction was conducted on compound **2** using dimethylformamide as the electrophile to afford 2,4-dimethoxy-6-pentylbenzaldehyde (**3**). The demethylation step was carried out using AlCl_3_ to afford 2,4-dihydroxy-6-pentylbenzaldehyde (**4**) (Scheme 1). 

The confirmation of the chemical structure and the selectivity of compound **4** was achieved through ^1^H-NMR analysis. In the spectrum, the two aromatic *meta* hydrogens exhibited different chemical shifts with a double doublet resonated at 6.24 and 6.22 ppm, each with a 4 Hz ^4^J-coupling. The aldehyde proton (H-C=O) of **4** displayed a distinct singlet resonance at 10.07 ppm, while one of the phenolic hydrogens exhibited a sharp singlet at a high chemical shift of 12.42 ppm (*ortho* position to the aldehyde group), and the second phenolic hydrogen exhibited a broad singlet resonated at 5.78 ppm. The spectrum closely resembled the one previously reported by Kim et al. [40]. Finally, CBD-aldehyde (**5**) was obtained by electrophilic aromatic substitution reaction between compound **4** and (+)-(1*R*,4*R*)-trans-p-mentha-2,8-dien-1-ol, achieving approximately 45% yield. ^1^H-NMR analysis of compound **5** revealed a singlet peak corresponding to the aromatic hydrogen resonated at 6.20 ppm. Additionally, the aldehyde hydrogen and *ortho* phenolic hydrogen appeared as two singlets resonated at 10.02 and 12.77 ppm, respectively. 

**Scheme 2:**
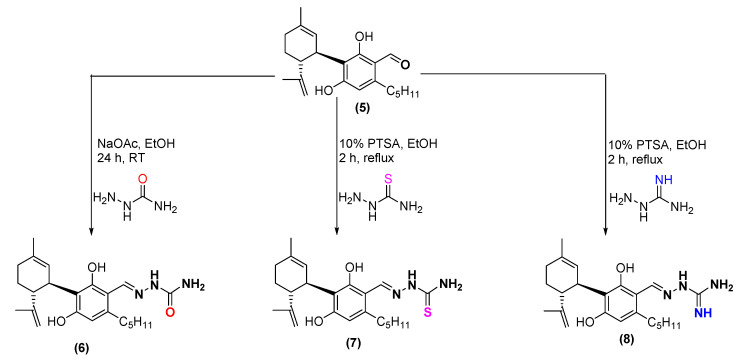
Chemical structures of semicarbazone-CBD-aldehyde (**6**), thiosemicarbazone-CBD-aldehyde (**7**), and aminoguanylhydrazone-CBD-aldehyde (**8**).

The synthesis of semicarbazone-, thiosemicarbazone-, and aminoguanylhydrazone- CBD-aldehyde derivatives (**6**, **7**, and **8**, respectively) were successfully achieved using the standard method under specific reaction conditions, yielding moderate-to-good yields (Scheme 2). 

The confirmation of the chemical structure of the hydrazone compounds was achieved through spectroscopic analysis. In ^1^H-NMR spectra, the imine proton (H-C=N) exhibited a characteristic singlet resonance at 8.26 and 8.57 ppm across all the derivatives. In compound **5**, the aldehyde proton (H-C=O) displayed a singlet resonance at 9.97 ppm. Regarding the NH_2_ protons, the semicarbazone and aminoguanylhydrazone derivatives resonated 6.30 and 5.44 ppm, respectively, as a broad singlet with indistinguishable protons. This NH_2_ group in the thiosemicarbazone derivative appears as two singlets resonated at 7.91 and 8.08 ppm, demonstrating that the protons were magnetically different because of the restricted rotation of the N-C bond. 

### 2.2. Antioxidant Activity by FRAP and DPPH Assays

**Figure 4:**
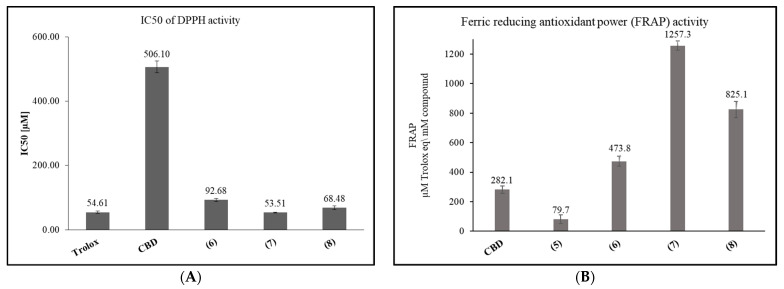
(**A**) IC_50_ of the tested compounds in DPPH radical scavenging activity. IC_50_ values are expressed as mean ± SD of the three independent experiments in triplicates. (**B**) Ferric-reducing antioxidant power (FRAP) assays for estimating antioxidant activities of the tested compounds (**5**, **6**, **7, 8** and CBD). Data are presented in trolox equivalent (TE) (1 mM of the tested compound is equivalent to X µM (values shown as bars) of trolox).

We determined the antioxidant properties of CBD, semicarbazone-CBD-aldehyde (**6**), thiosemicarbazone-CBD-aldehyde (**7**), and aminoguanylhydrazone-CBD-aldehyde (**8**) using two methods: FRAP and DPPH. The analysis revealed strong reducing activity of the newly synthesized compounds (**6**, **7**, and **8**) in both assays. The antioxidant activity of the compounds was compared to the trolox activity. In the 1,1-diphenyl-2-picryl-hydrazyl (DPPH), IC_50_ values were 54.61 μM for trolox and 92.68, 53.51, 68.48, and 506.10 μM for **6**, **7**, **8**, and CBD, respectively (Figure 4A). IC_50_ corresponds to the concentration of tested compounds that is able to scavenge 50% of the initial DPPH radicals. Low IC_50_ values indicate high antioxidant activity [41]. Compound **7** shows the highest DPPH radical-scavenging activity, which is similar to the activity of the trolox. Compounds **7** and **8** show higher scavenging activity than compound **6**. However, compound **6** still exhibits much higher activity than that of CBD (Figure 4A). 

The ferric-reducing antioxidant potential (FRAP) assay is a simple and inexpensive protocol that is particularly helpful in assessing the antioxidant power of a sample in which those constituents are present that act by reducing ions or by donating an electron and not by the radical quenching mechanism [42]. The higher the FRAP value, the greater the antioxidant activity. Compound **7** exhibits remarkable antioxidant activity detected by the FRAP method (1257 μM TE), while **6** and **8** exhibit lower activity with values of 473 and 825 μM TE, respectively, which is still about 2-fold more than the FRAP activity of CBD (282.1 μM TE) (Figure 4B). 

### 2.3. Antioxidant Activity by LDL Oxidation

**Figure 5:**
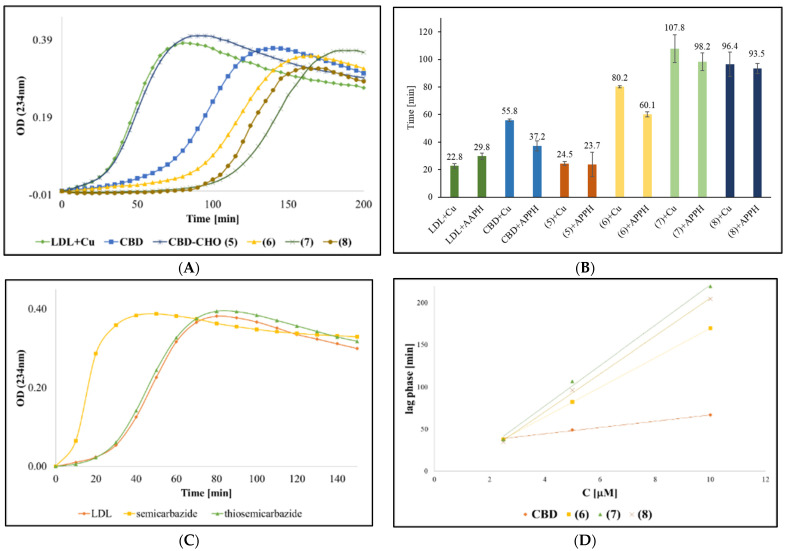
(**A**) Effect of the tested compounds (CBD, 5, 6, 7, and 8) on the oxidation of LDL induced by Cu^2+^ (control: LDL+Cu^2+^). The concentration of each compound is 5 µM (0.2% DMSO). (**B**) Effect of the tested compounds on the lag time values. (**C**) Effect of semicarbazide and thiosemicarazide (5 µM) on the oxidation of LDL induced by Cu^2+^. (**D**) Linear regression of lag time versus different concentrations of the tested compounds (2.5, 5, and 10 µM). Conjugated dienes formation during copper-mediated LDL oxidation was measured by determining the absorbance at 234 nm in intervals of 5 min at 37 °C. Each experiment was repeated in three independent experiments in duplicates. All the results show significant value relative to the control (LDL without tested compounds) (*p* ≤ 0.001) CBD: Cannabidiol. (**6**): Semicarbazone- CBD-aldehyde. (**7**): Thiosemicarbazone-CBD-aldehyde. (**8**): aminoguanylhydrazone-CBD-aldehyde.

The kinetic formation of conjugated dienes (CDs) during AAPH and Cu^2+^-induced LDL oxidation was examined. Oxidation of lipids in LDL is suggested to be a radical reaction that proceeds through three main phases: initiation, propagation, and termination. The kinetic profile of LDL oxidation is characterized by the lag time (initiation) and the oxidation rate (propagation phase). A representative kinetic analysis of LDL oxidation induced by Cu^2+^ is shown in Figure 5, and the oxidation parameters are summarized in Table 1. The LDL oxidation lag time significantly increased from 22.76 min to 55.81, 80.17, 107.84, and 96.4 min after incubation with CBD, **6**, **7**, and **8**, respectively. However, there wasn’t a significant difference in the oxidation rate among the compounds tested (approximately 30% inhibition). It’s noteworthy that incubating LDL with either compound **5** or semicarbazide/thiosemicarbazide did not result in any observable effect on its oxidation (Figure 5A,C).

**Figure 6:**
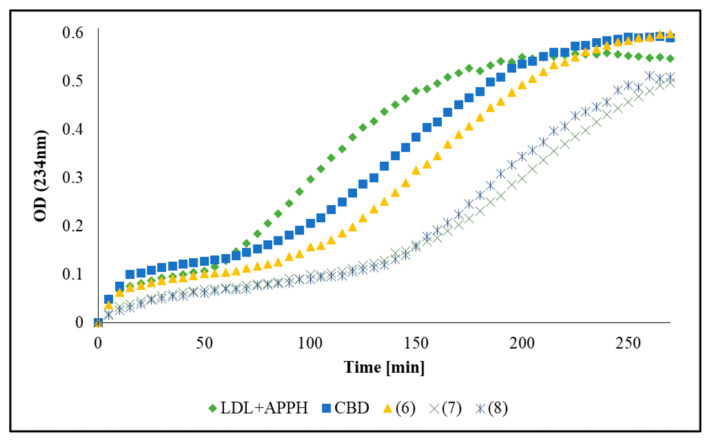
Effect of the synthesized compounds on the oxidation of LDL induced by AAPH. The concentration of each compound is 5 μM (0.2% DMSO). Conjugated diene formation during copper-mediated LDL oxidation was monitored for 200 min for changes in absorbance at 234 nm. Each experiment was repeated in three independent experiments in duplicates.

LDL oxidation kinetics induced by 1 mM AAPH are illustrated in Figure 6 and Table 2 in the absence and presence of the tested compounds. CBD was almost ineffective in protecting LDL from AAPH-mediated oxidation (lag time 37.24 min versus 29.79 min). However, the lag time of LDL oxidation after incubation with **6**, **7**, and **8** was significantly increased from 29 min to 60.09, 98.20, and 93.5 min, respectively. In addition, LDL oxidation was inhibited by about 19% after incubation with compounds **6**, **7**, **8**, and CBD (Table 2). 

### 2.4. Effect of Synthesized Compounds on LDL Tryptophan Fluorescence

**Figure 7:**
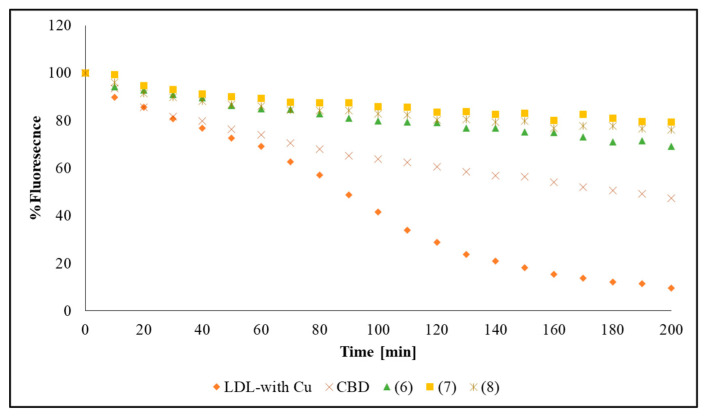
Kinetics of LDL-tryptophan fluorescence during copper-induced oxidation. LDL was incubated in PBS with 10 μM CuSO_4_ in the absence or the presence of 5 μM (0.2% DMSO) of the tested compounds. Fluorescence (Ex/Em = 285/332 nm) was measured at intervals of 10 min at 37 °C. Each experiment was repeated in three independent experiments in duplicates.

The fluorescence spectra of native LDL display a single band at approximately 332 nm, which is assigned to the tryptophan (Trp) residues in ApoB-100 [43,44]. Loss of the tryptophan fluorescence is a marker for oxidations at the protein core of LDL. The loss of the Trp fluorescence in LDL induced by Cu^2+^ is shown in Figure 7. When LDL was incubated with compounds **6**, **7**, and **8**, there was a significant increase in the t_1/2_ of Trp quenching. After 200 min, Trp fluorescence was quenched by approximately 70% after incubation with **6**, **7**, and **8**. CBD, on the other side, was able to quench the Trp fluorescence by 50% after the same time (Figure 7). 

## 3. Discussion

Nowadays, developing new bioactive compounds from natural compounds has become an interesting area. Over the past decades, there has been a significant interest in synthesizing novel cannabinoid derivatives and evaluating their biological activities. Among these, CBD, a cannabis-derived compound, has received considerable interest, and its chemical framework has become increasingly attractive for medicinal chemists [45,46]. In the current research, we focused on modifying the CBD framework at the 3-position of the olivetolic acid, where findings indicate that modifying CBD at this position could be a promising strategy for developing new cannabinoid compounds based on the CBD framework [25,45,47]. 

To prepare the desired conjugated molecules, it was necessary to synthesize the 3-formyl cannabidiol compound (**5**) and then apply condensation reactions between the aldehyde group and semicarbazide derivatives. Initial attempts to prepare compound **5** directly from cannabidiol using methods such as the Vilsmeier-Haack reaction and Reimer-Tiemann formylation were unsuccessful. Consequently, we chose to begin the synthesis from 1-bromo-3,5-dimethoxybenze, as illustrated in Scheme 1. Different research groups have reported the synthesis of compound **4** directly from olivetol using the Vilsmeier-Haack reaction [26,40,48]. It is worth noting that our synthesis approach can be utilized not only for compound **4** but also for preparing various resorcinol-based compounds with different alkyl chains.

Upon successful completion of the total synthesis of **5**, we synthesized and characterized (thio)semicarbazone- and aminoguanidinehydrazone-CBD-aldehyde compounds (**6**, **7**, and **8**).

The antiradical- and ferric-reducing potentials of the prepared compounds were investigated. A comparative study was conducted using trolox as the standard. The data clearly demonstrate the remarkable antioxidant potential of the newly synthesized compounds. Compound **7** exhibited the highest antioxidant potential, similar to trolox, with the lowest IC_50_ of 53 μM and 1257 μM TE/mM, respectively, for DPPH radical scavenging activity and FRAP assays. These findings strongly indicate that incorporating Schiff base moieties onto the CBD scaffold significantly enhances antioxidant properties through radical scavenging or ferric-reducing activities. Interestingly, introducing a sulfur atom within the semicarbazone motif appears to result in superior activity compared to derivatives based on nitrogen and oxygen (Figure 4). CBD-aldehyde (**5**) showed limited activity in both the FRAP assay, with 79.7 μM TE (Figure 4B), and the DPPH assay, where it exhibited an IC_50_ > 1500 μM. 

The oxidation of LDL and apolipoprotein B-100 (ApoB-100) play a critical role in atherogenesis and are oxidized by exposure to trace metal ions, reactive oxygen species (ROS), lipoxygenase, and others [44,49]. The oxidation process can lead to the formation of foam cells, which is responsible for the build-up of atherosclerotic plaques [50]. Thus, reducing the formation of the oxLDL is of great importance in the prevention of atherosclerosis and heart-related disease [51]. Lipid oxidation can be easily followed by measuring the kinetic profile of the formation of conjugated dienes [52], which is often used to characterize the atherogenicity of LDL or the therapeutic usefulness of antioxidants. We examined the ability of the synthesized compounds to prevent LDL oxidation induced either by CuSO_4_ or AAPH. The use of ‘catalytic’ copper ions to promote peroxidation of LDL under in vitro conditions can be considered a valid model to study events occurring in the atherosclerotic arterial wall [53]. Alternatively, AAPH, a hydroxyl radical generator, consistently produces peroxyl radicals in an aqueous medium, leading to the chain oxidation of human LDL through a free radical-mediated mechanism [54]. APPH is also considered an effective alternative for mimicking the oxidative damage to lipid and protein components of LDL during the atherogenic process. Our results showed that the synthesized compounds were able to reduce the formation of conjugated dienes and significantly increase the lag time in a concentration-dependent manner (Figure 5D). When compound **7** was incubated with LDL, the lag time was increased from 25 min to 107 min and 98 min by copper ions and AAPH, respectively, and inhibited the LDL oxidation by ~35%. 

On the other hand, when compound **6** was incubated with LDL, the lag time was increased from 25 min to 80 min and 60 min by copper ions and AAPH, respectively, and inhibited the LDL oxidation by ~18%. It is worth noting that incubation of LDL with compound **5**, or (thio)-semicarbazide precursor compounds, did not have any effect on its oxidation but rather showed moderate pro-oxidant activity of the kinetic profile of the LDL oxidation (Figure 5C). The lower antioxidant activity of **5** is most probably due to the strong formation of intramolecular hydrogen bonds between the phenolic hydrogen and adjacent aldehyde moiety to afford a thermodynamically stable six-membered conjugated ring. Strong evidence for this hypothesis was shown in the ^1^H-NMR analysis, where the phenolic hydrogen appears in a high chemical shift that resonated at 12.77 ppm [55,56]. 

Copper ion (Cu^2+^) is able to complex with proteins, including ApoB-100; the oxidation of the LDL induced by Cu^2+^ is accompanied by loss of Trp fluorescence, and it’s assumed that a finite number of Cu^2+^ ions can bind to ApoB-100 in close vicinity to Trp residues and cause its oxidation and degradation [44,52]. Therefore, we assume that the protective effect of our synthesized compounds on the copper-mediated oxidation may be due to their capacity to interact and coordinate some copper binding sites in LDL protein and, as a result, decrease LDL susceptibility to copper-catalyzed oxidation. This is confirmed by monitoring the LDL-Trp fluorescence kinetic profile, in which compounds **6**, **7**, and **8** have a greater capacity to protect the degradation of amino acid residues resulting from Cu^2+^ complexation than CBD. Our results showed an increase in the t_1/2_ of Trp-fluorescence in Cu^2+^-oxidized LDL when the synthesized compounds were present, compared to the t_1/2_ in the absence of these compounds or the presence of CBD (Figure 7).

The protective effect of the new derivatives against LDL oxidation can occur via several mechanisms, such as (1) chelating free Cu^2+^ ions to form redox-inactive complexes and thus reducing metal-catalyzed oxidation of LDL. The proposed complex features a tridentate XNO-coordinating ligand (2) inhibiting the binding of Cu^2+^ ions to ApoB-100, which subsequently prevents the degradation of amino acid ApoB-100 residues (3) scavenging of free radicals and chain breaking of the initiation stage.

## 4. Materials and Methods

### 4.1. General

All chemicals and reagents were purchased from Sigma-Aldrich, Rehovot, Israel. Anhydrous tetrahydrofuran was dried over sodium and used freshly. Flash column chromatography was performed with Merck ultra-pure silica gel (230–400 mesh). Yields refer to isolated compounds greater than 95% purity as determined by proton nuclear magnetic resonance spectroscopy (NMR, 400 MHz, Bruker Corporation, Billerica, MA, USA), high-performance liquid chromatography (HPLC, Agilent 1290, Agilent Technologies, Inc., Santa Clara, CA, USA), and gas chromatography (GC, Agilent 7890A, Agilent Technologies, Inc., Santa Clara, CA, USA) analysis. LDL was isolated and purified from commercially available human serum using the manufacturer’s recommended protocol [57]. For complete analytical data, please refer to the Supplementary Materials (Tables S1–S29).

### 4.2. Preparation of 1,3-Dimethoxy-5-pentylbenzene (**1**) [58]

A solution of 1-bromo-3,5-dimethoxybenzene (46 mmol, 10 g) in 30 mL dry THF was added dropwise to a stirred slurry of magnesium turnings (46 mmol, 1.1 g) in freshly distilled tetrahydrofuran (THF) (50 mL), keeping the temperature between 30 and 40 °C. After completion of the addition, the solution was refluxed until full dissolution of the magnesium turnings. The reaction mixture was then then cooled down to 25 °C. A solution of bromopentane (55 mmol, 8.33 g), lithium bromide (2.09 mmol, 0.182 g), and CuCl_2_ (2.23 mmol, 0.3 g) in 20 mL THF was added dropwise, and then the solution was refluxed and held overnight.

The solution was cooled down to 5 °C, and 100 mL of 10% acetic acid was added dropwise. The mixture was extracted three times with ethyl acetate, and the organic layer was washed with 5% sodium bicarbonate to achieve a final pH of ca. 8. The organic phase dried over Na_2_SO_4_, filtered, and concentrated under reduced pressure. The crude was chromatographed on silica gel using 5% of ethyl acetate in hexane as the eluent to afford 5 g of 1,3-dimethoxy-5-penylbenzene (**1**) as a yellowish oil (52% yield). GCMS purity > 97%. GC-MS (*m*/*z*): 208.15 (M), 193.2 (M-methyl), 179.1 (M-ethyl), 152.1 (M-butyl). 

### 4.3. Preparation of 2-Bromo-1,5-dimethoxy-3-pentylbenzene (**2**)

*N*-bromosuccinimide (0.024 mole, 4.27 g) in small portions was added to a stirred solution of 1,3-dimethoxy-5-pentylbenzene (0.024 mole, 5 g) in 100 mL of dichloromethane, while maintaining the temperature below 10 °C. After completion of the addition, the reaction mixture held for 30 min at room temperature (RT). Then, the reaction mixture was filtered, and the filtrate was washed with 5% sodium bicarbonate and extracted with dichloromethane. The organic phase was dried over Na_2_SO_4_, filtered, and concentrated under reduced pressure to generate compound **2** as a colorless oil with quantitative yield. GCMS purity > 96%. GC-MS (*m*/*z*): 286.1, 288.05 (M, two isotopes 1:1), 228.0, 230.0 (M-butyl, two isotopes 1:1), 207.2 (M-Br). 1H-NMR (DMSO-d_6_, 400 MHz): 6.23 (2H, s), 3.81 (3H, s, CH_3_O), 3.76 (3H, s, CH_3_O), 2.65 (2H, t), 1.53 (2H, m), 1.31 (4H, m), 0.86 (3H, t). ^13^C-NMR (DMSO-d_6_), δ: 164.54, 161.48, 148.47, 112.30, 108.76, 102.62, 61.41, 60.56, 41.08, 36.26, 34.38, 27.18, 19.11. 

### 4.4. Preparation of 2,4-Dimethoxy-6-pentylbenzaldehyde (**3**)

A solution of 2-bromo-1,5-dimethoxy-3-pentylbenzene (6.9 mmol, 2 g) in 30 mL anhydrous THF was added dropwise to a stirred slurry of magnesium turnings (6.9 mmol, 0.17 g) in anhydrous THF (20 mL), keeping the temperature between 30 and 40 °C. After completion of the addition, the solution was refluxed until the magnesium turnings were fully dissolved. The reaction mixture was then cooled down to 25 °C, and an excess of *N,N*-dimethylformamide (5 mL) was added dropwise, and then the solution was refluxed for 1 h. 

The solution was cooled down to 5 °C, and 1 M HCl (50 mL) was added dropwise. The mixture was extracted three times with ethyl acetate, and the organic layer was washed with 5% sodium bicarbonate to achieve a final pH of ca. 8. The organic phase was concentrated under reduced pressure and purified with column chromatography using 10% ethyl acetate in hexane as the eluent to afford 1.2 g of 2,4-dimethoxy-6-pentylbenzaldehyde (**3**). 1HNMR (CDCl3, 400 MHz), δ: 10.39 (1H,s), 6.25 (2H, dd), 3.80 (2H, s), 3.79 (3H, s), 2.87 (2H, dd), 1.46 (2H, m), 1.29 (4H, m), 0.82 (3H, t). ^13^C-NMR (CDCl3), δ: 192.28, 165.37, 164.53, 149.70, 116.78, 108.06, 95.66, 55.76, 55.42, 34.57, 31.97, 30.98, 22.59, 14.09. GCMS purity > 96%. 

### 4.5. Preparation of 2,4-Dihydroxy-6-pentylbenzaldehyde (**4**) 

A solution of 2,4-dimethoxy-6-pentylbenzaldehyde (8 mmol, 1.88 g) in dichloromethane (50 mL) was added dropwise to a slurry of AlCl_3_ (19.9 mmol, 2.66 g) in dichloromethane (50 mL), while maintaining the temperature below 30 °C. The reaction mixture was stirred at room temperature for 24 h. The reaction was quenched with 50 mL of 1M HCl in ice and extracted three times with dichloromethane. The organic phase was washed with 5% sodium bicarbonate and concentrated under reduced pressure, and treated with hexane, then filtered to afford 1.40 g of brown solid (85%). ^1^HNMR (CDCl_3_, 400 MHz), δ: 12.12 (1H, s), 10.07 (1H, s), 6.23 (1H, d), 6.22 (1H, d), 5.7 (1H, bs), 2.81 (2H, t), 1.63 (2H, m), 1.35 (4H, m), 0.92 (3H, t). ^13^C-NMR (CDCl3), δ: 193.02, 166.45, 164.49, 150.73, 112.53, 110.32, 32.23, 31.92, 31.59, 22.46, 13.98. HR-MS (ESI+) *m*/*z* = 209.1175 (M+). HPLC purity > 97%.

### 4.6. Synthesis of 3-Formyl Cannabidiol (CBD-Aldehyde) (**5**)

*Para*-toluene sulfuric acid monohydrate (PTSA, 2.4 mmol, 0.46 g) was added to a stirred solution of 2,4-dihydroxy-6-pentylbenzaldehyde (24 mmol, 5 g) in dichloromethane (100 mL) and cooled to 5 °C using an ice bath. To this solution was added a cold solution of (+) -(1R,4R)-trans-p-mentha-2,8-dien-1-ol (24 mmol, 3.65 g) in dichloromethane (25 mL). After stirring for 1 h at this temperature, the reaction mixture was quenched with saturated sodium bicarbonate and extracted 3 times in dichloromethane. The organic phase was washed with 1 M sodium hydroxide, followed by washing with 1 M HCl. The organic phase was dried over anhydrous Na_2_SO_4_, filtered, and evaporated. The residue was subjected to chromatography in a silica gel column eluted with 5% ethyl acetate in hexane to produce 3.6 g of yellowish oil (45% yield). 

The product was identified by LC-MS, GCMS, and NMR analyses. ^1^H-NMR (CDCl_3_, 400 MHz), δ: 12.92 (1H, s), 10.52 (1H, s), 9.97 (1H,s), 6.24 (1H,s), 5.10 (1H,s), 4.85 (1H, m), 4.44 (1H, m), 3.86 (1H, m), 2.81 (2H, t), 2.16 (1H, m), 2.02 (2H, m), 1.73 (2H, m), 1.67 (3H, s), 1.64 (3H, s), 1.58 (2H, m), 1.35 (4H, m), 0.92 (3H, t). ^13^C-NMR (CDCl_3_), δ: 192.81, 164.30, 162.89, 147.35, 146.99, 140.74, 123.60, 114.47, 111.83, 111.47, 110.56, 46.76, 34.53, 32.23, 31.65, 31.61, 30.28, 23.73, 22.47, 13.99. FT-IR (υ, cm^−1^): 3675, 1618, 1393. $\alpha_{598\;nm}^{25\;{}^{\circ}C}\left(EtOH\right)=-91.52$ . HR-MS (ESI^+^) *m*/*z* = 343.2269 (M^+^). HPLC purity > 96%. 

### 4.7. Synthesis of Aminoguanylhydrazone and (Thio)-Semicarbazone Cannabidiol-Aldehyde Derivatives

Semicarbazide hydrochloride salt (1.1 equiv.), CBD-aldehyde (1 equiv.), and sodium acetate (NaOAc, 1.2 equiv.) were dissolved in 5 mL of absolute ethanol. The reaction mixture was stirred at RT under an inert atmosphere overnight. The reaction completion and formation of the product were confirmed using thin-layer chromatography. The solvent was then evaporated to dryness. The residue was extracted with dichloromethane and saturated bicarbonate solution, dried over sodium sulfate, filtered, and evaporated to dryness. 

3-semicarbazone-CBD-aldehyde (**6**) was purified in chromatography using a silica gel column eluted with 20% ethyl acetate in hexane to give an off-white solid (60% yield). The product was identified by LC-MS and NMR analyses. ^1^H-NMR (DMSO-d_6_, 400 MHz), δ: 11.52 (1H, bs), 10.11 (1H, s), 9.67 (1H,bs), 8.36 (1H, s), 6.30 (2H, bs), 6.25 (1H, s), 5.20 (1H, s), 4.60 (1H, s), 4.52 (1H, s), 4.03 (1H, d), 3.22 (1H, t), 2.61 (3H, m), 2.21 (1H, m), 2.08 (1H, m), 1.71 (8H, m), 1.54 (2H, m), 1.38 (4H, m), 0.98 (3H, t). ^13^C-NMR (DMSO-d_6_), δ: 155.97, 149.39, 143.36, 140.98, 138.29, 130.96, 126.70, 125.93, 115.36, 110.31, 108.81, 43.81, 35.92, 32.74, 31.66, 31.50, 31.44, 30.74, 29.87, 23.77, 22.55, 22.46, 19.61, 14.44. FT-IR (υ, cm^−1^): 3675, 1682, 1623, 1574. $\alpha_{598\;nm}^{25\;{}^{\circ}C}(EtOH)=-88.96$ . mp = 106 °C. HR-MS (ESI^+^) *m*/*z* = 400.2599 (M^+^). HPLC purity > 96%. 

Aminoguanidine or thiosemicarbazide hydrochloride salts (1.1 equiv.), CBD-aldehyde (1 equiv.), and PTSA (10% mole) were dissolved in 5 mL of absolute ethanol. The reaction mixture was refluxed for 2 h under an inert atmosphere. The reaction completion and formation of the product were confirmed using thin-layer chromatography. The solvent was then evaporated to dryness. The residue was extracted with dichloromethane and saturated bicarbonate solution, dried over Na_2_SO_4_, filtered, and evaporated to dryness. 

3-thiosemicarbazone-CBD-aldehyde **(7)** was purified in chromatography using a silica gel column eluted with 15% ethyl acetate in hexane to give a white solid (65% yield). The product was identified by LC-MS and NMR analyses. ^1^H-NMR (DMSO-d_6_, 400 MHz), δ: 11.21 (1H, s), 10.39 (1H, bs), 9.67 (1H, bs), 8.57(1H, s), 8.08 (1H, bs), 7.91 (1H, bs), 6.24 (1H, s), 5.16 (1H, s), 4.56 (1H, s), 4.47 (1H, s), 3.98 (1H, d), 3.17 (1H, t), 2.57 (2H, m), 2.19 (1H, m), 2.02 (1H, m), 1.71 (8H, m), 1.50 (2H, m), 1.36 (4H, m), 0.93 (3H, t). ^13^C-NMR (DMSO-d_6_), δ: 177.89, 170.79, 149.30, 146.82, 142.63, 131.13, 126.52, 115.44, 110.44, 107.42, 43.75, 35.92, 32.93, 31.77, 31.71, 30.74, 29.84, 23.76, 22.46, 21.22, 19.56, 14.45. FT-IR (υ, cm^−1^): 3450, 1586, 1506, 1258. $\alpha_{598\;nm}^{25\;{}^{\circ}C}(EtOH)=-97.08$ . mp = 122 °C. HR-MS (ESI^+^) *m*/*z* = 416.2371 (M^+^). HPLC purity > 97%. 

3-aminoguanylhydrazone-CBD-aldehyde (**8**) was purified in chromatography using a silica gel column eluted with 5% methanol in dichloromethane to give a white solid (85% yield). The product was identified by LC-MS and NMR analyses. ^1^H-NMR (DMSO-d_6_, 400 MHz), δ: 12.63 (1H, bs), 9.20 (1H, bs), 8.30 (1H, s), 6.10 (1H, s), 5.33 (4H, m) 5.12 (1H, s), 4.52 (1H, s), 4.42 (1H, s), 3.93 (1H, d), 3.13 (1H, t), 2.61 (3H, m), 2.21 (1H, m), 2.08 (1H, m), 1.71 (8H, m), 1.54 (2H, m), 1.38 (4H, m), 0.98 (3H, t).^13^C-NMR (DMSO-d_6_), δ: 157.71, 149.94, 147.78, 140.52, 138.24, 130.88, 128.86, 126.88, 125.94, 115.09, 110.29, 108.82, 43.79, 35.95, 32.64, 31.53, 30.77, 29.94, 23.77, 22.46, 19.67, 14.42. FT-IR (υ, cm^−1^): 3362, 1586, 1557, 1435. $\alpha_{598\;nm}^{25\;{}^{\circ}C}(EtOH)=-90.54$ . mp = 110 °C. HR-MS (ESI^+^) *m*/*z* = 399.2758 (M^+^). HPLC purity > 98%. 

### 4.8. Free Radical 1,1-Diphenyl-2-picryl-hydrazyl (DPPH)-Scavenging Assay

The total antioxidant capacity of the synthesized compounds was determined using the DPPH radical as a reagent, according to the procedure described by Chang et al. with some modifications [59]. Briefly, 100 μL of 100 μM solution of DDPH in ethanol was added to 10 μL of the synthesized compounds at different concentrations (500, 250, 125, 62.5, 31.25, 15.62, and 7.81 μM). Trolox, a well-known standard with strong antioxidant activities, was used as a positive control; the absorbance was measured at 517 nm after 30 min of incubation at room temperature in the dark, using a Tecan Infinite 200 PRO plate reader. The percentage of the inhibition of DPPH oxidation was calculated according to the following formula:

$$DPP{H_{scavenging}}_{effect\left(\%\right)}=\frac{A_{control}-A_{sample}}{A_{control}}\times 100$$

where $A_{control}$ means the absorbance of the control sample and $A_{sample}$ means the absorbance of the standard or tested compound. The results were also expressed as the concentration needed to inhibit a biological process by 50% values (IC_50_).

### 4.9. Ferric-Reducing Antioxidant Power (FRAP) Assay

The antioxidant capacity of the synthesized compounds was evaluated by the reduction of ferric 2,4,6-tripyridyl-s-triazine complex (Fe^3+^-TPTZ) to the ferrous form (Fe^2+^-TPTZ) at low pH, which causes a colored ferrous-tripyridyltriazine complex to form, according to the procedure described by Benzie et al., with some modifications [42]. Briefly, 20 µL of the tested compound was mixed with 130 µL FRAP reagent (buffer acetate pH = 3.6, 10 mM TPTZ, 40 mM HCl, 20 mM FeCl_3_/H_2_O ratio 10:1:1) and then incubated for 45 min at 37 °C. FRAP values are obtained by comparing the absorbance change at 595 nm in test reaction mixtures with those containing ferrous ions in known concentrations using trolox as a calibration curve (concentrations between 7.8 and 500 µM). The results were expressed as μM of trolox equivalent (TE). The absorbance was measured using a Tecan Infinite 200 PRO plate reader.

### 4.10. AAPH and Cu^2+^-Induced LDL Oxidation

The oxidation was monitored by measuring the increase in absorbance at 234 nm, according to the procedure described by Esterbauer et al. [60] Oxidation of LDL was carried out in a UV ELISA plate at 37 °C and measured using a Tecan Infinite 200 PRO plate reader. A total of 77 μL phosphate buffer saline (PBS), 3 μL LDL (final concentration of 50 μg protein/mL in PBS pH 7.4), and 10 μL of the tested compounds were added to each well. After 5 min, the oxidation was initiated by the addition of 10 μL CuSO_4_ (10 µM) or 2,2-azobis-(2-amidinopropane dihydrochloride) (AAPH, 1 mM). LDL oxidation was determined by continuous monitoring (every 5 min for 200 min) of conjugated diene formation at 234 nm. The lag time and rate constant (K) of the propagation phase were calculated by nonlinear regression. Percent inhibition of LDL oxidation was calculated as: 100 − (K(LDL + sample)/K(LDL) × 100).

### 4.11. Measurement of LDL-Tryptophan Fluorescence

Tryptophan fluorescence was measured in a solution of 3 μL of LDL (50 μg protein/mL) in 77 μL PBS (pH 7.4), according to the procedure described by Giessauf et al. [44]. The kinetic of LDL oxidation was followed by measuring the decrease of the Trp-fluorescence after the addition of 10 μL of CuSO_4_ (10 μM) in the absence or presence of the tested compounds (10 μL) (excitation at 285 nm and emission at 332 nm). Fluorescence was measured every 10 min. Data are shown as a percentage of Trp-fluorescence decrease in each sample. The time required for reaching half Trp-fluorescence (t_1/2_) was calculated. 

### 4.12. Statistical Analysis

Data are expressed as mean ± SD. Statistical analysis was performed using a one-way analysis of variance. Linear regression was performed to identify a possible dose-dependent effect. Values of *p* < 0.05 were considered significant.

## 5. Conclusions

In conclusion, we have successfully synthesized and characterized novel aminoguanylhydrazone- and (thio)-semicarbazone-based cannabidiol compounds. These compounds are classified as organic compounds characterized by the presence of an imine (–N = CH-) group, known as Schiff bases or hydrazones, and more specifically, they are known as semicarbazones (–C=N-NH(C=X)-NH_2_). We conducted assessments of their antioxidant properties using various assays: DPPH, FRAP, and evaluation of LDL oxidation induced by Cu^2+^ and AAPH. The results obtained demonstrated the ability of the newly developed compounds to protect against LDL oxidation induced by AAPH and copper ions. Notably, thiosemicarbazone-CBD-aldehyde (**7**) demonstrates the most potent compound among the synthesized derivatives. However, further investigations are necessary to elucidate the precise molecular mechanisms underlying their activity in cell culture models and understand their structural indications and receptor–ligand interactions. These studies will provide valuable insights into how these structural modifications can potentially enhance binding. Addressing these crucial aspects is an active area of research in our laboratory, and we look forward to advancing our understanding of these promising compounds.

## Figures and Tables

**Table 1:** Effect of the tested compounds on lag time, oxidation rate of the reaction propagation, and % inhibition of LDL oxidation induced by Cu^2+^.

Compound	Lag Time [min] ± SD	Oxidation Rate	%Inhibition
LDL	22.76 ± 1.7	0.0081	0.0
CBD	55.81 ± 1.3	0.0052	36.07
**(5)**	24.52 ± 0.8	0.0080	1.64
**(6)**	80.17 ± 0.6	0.0053	35.25
**(7)**	107.84 ± 10.1	0.0058	29.30

**Table 2:** Effect of the tested compounds on lag time, oxidation rate of the reaction propagation, and % inhibition of LDL oxidation induced by AAPH.

Compound	Lag Time [min] ± SD	Oxidation Rate	%Inhibition
LDL	29.79 ± 2.2	0.0042	0.0
CBD	37.24 ± 3.7	0.0034	19.05
**(5)**	60.09 ± 1.8	0.0035	16.67
**(6)**	98.23 ± 6.4	0.0034	19.05
**(7)**	87.25 ± 5.3	0.0034	19.05

## Data Availability

The original contributions presented in the study are included in the article/Supplementary Materials, further inquiries can be directed to the corresponding author.

## Supplementary Materials

The following supporting information can be downloaded at: https://www.mdpi.com/article/10.3390/ijms25179579/s1.
