# Correction: Peretz, E.; Musa, S. Design, Synthesis, and Characterization of Novel Cannabidiol-Based Derivatives with Potent Antioxidant Activities. *Int. J. Mol. Sci.* 2024, *25*, 9579

**DOI:** 10.3390/ijms26051927

**Published:** 2025-02-24

**Authors:** Eliav Peretz, Sanaa Musa

**Affiliations:** 1 Department of Biotechnology, Tel-Hai Academic College, Kiryat Shmona 11016, Israel; 2 Natural Compounds and Organic Synthesis Laboratory, Migal-Galilee Research Institute, Kiryat Shmona 11016, Israel

In the original publication, there was a mistake in Figure 5D as published. In the original version, Figure 5D was mistakenly identical to Figure 5B [1]. It should have been a graph representing the linear regression of lag time versus varying concentrations of the tested compounds (2.5, 5, and 10 µM). The correct version of Figure 5D appears below. The authors state that the scientific conclusions are unaffected. This correction was approved by the Academic Editor. The original publication has also been updated.

## Figures and Tables

**Figure 5:**
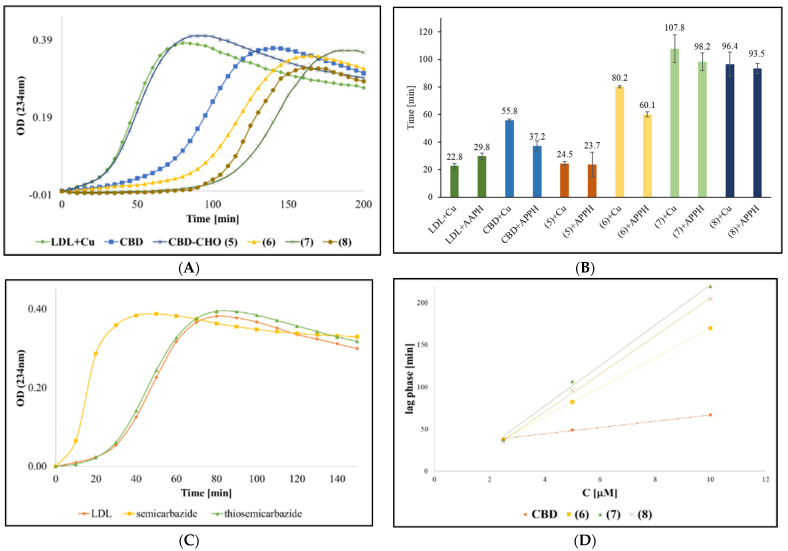
(**A**) Effect of the tested compounds (CBD, 5, 6, 7, and 8) on the oxidation of LDL induced by Cu^2+^ (control: LDL+Cu^2+^). The concentration of each compound is 5 µM (0.2% DMSO). (**B**) Effect of the tested compounds on the lag time values. (**C**) Effect of semicarbazide and thiosemicarazide (5 µM) on the oxidation of LDL induced by Cu^2+^. (**D**) Linear regression of lag time versus different concentrations of the tested compounds (2.5, 5, and 10 µM). Conjugated dienes formation during copper-mediated LDL oxidation was measured by determining the absorbance at 234 nm in intervals of 5 min at 37 °C. Each experiment was repeated in three independent experiments in duplicates. All the results show significant value relative to the control (LDL without tested compounds) (*p* ≤ 0.001) CBD: Cannabidiol. (**6**): Semicarbazone- CBD-aldehyde. (**7**): Thiosemicarbazone-CBD-aldehyde. (**8**): aminoguanylhydrazone-CBD-aldehyde.
