# Supplementary material for: Design, Synthesis, and Characterization of Novel Cannabidiol-Based Derivatives with Potent Antioxidant Activities
Source: Int J Mol Sci. 2024 Sep 4;25(17):9579. doi: 10.3390/ijms25179579 (PMC11395037; doi:10.3390/ijms25179579)
Supplement: Supplementary file 1 [file ijms-25-09579-s001.zip › ijms-3164600-supplementary.pdf]

Supporting information

Design, Synthesis, and Characterization of Novel Cannabidiol-Based Derivatives with Potent Antioxidant Activities

Eliav Peretz [1,2] and Sanaa Musa [1,2]*

[1] Department of Biotechnology, Tel-Hai Academic College, Kiryat Shmona 11016, Israel

[2] Natural Compounds and Organic Synthesis Laboratory, Migal-Galilee Research Institute, Kiryat Shmona 11016, Israel

* Correspondence: sanaa@migal.org.il or musasan@telhai.ac.il

General Considerations. All chemicals and reagents were purchased from Sigma-Aldrich. Anhydrous tetrahydrofuran was dried over sodium and used freshly. HPLC-grade acetonitrile and formic acid were used. Flash column chromatography was performed with Merck ultra-pure silica gel (230-400 mesh). Yields refer to isolated compounds greater than 95% purity as determined by proton Nuclear Magnetic Resonance spectroscopy (^{1}H-NMR), High-performance liquid chromatography (HPLC), and Gas chromatography (GC) analysis.

HPLC Analysis

HPLC analysis was performed with UHPLC connected to a photodiode array detector (Agilent 1290), with a reverse-phase column (Phenomenex RP-18, 150 X 4.6 mm, 3 µm). The mobile phase was a mixture of (A) double-distilled water (DDW) with 0.1% formic acid and (B) acetonitrile with 0.1% formic acid with a flow of 0.5ml/min and gradient starting with 5% B and increasing in a concentration to 95% B for 30 min and then kept at 95% B for an additional 5 min.

LC–MS Analysis

The LC-MS analysis was performed with a heated electrospray ionization (HESI-II) source connected to a Q Exactive™ Plus Hybrid Quadrupole-Orbitrap™ Mass Spectrometer (Thermo Scientific). The ESI capillary voltage was set to 3900 V, capillary temperature to 350°C, and gas temperature to 350°C. Nitrogen gas (N_2) was used. The MS conditions were set as follows: the flow rate of sheath gas, aux gas, and sweep gas was kept at 35 L/min, 10 L/min, and 1 L/min, respectively.

GC-MS analysis

The analysis of the compounds was performed using gas chromatogram instrument (Agilent 7890A), equipped with Phenomenex Zebron ZB-5 column (30m, 0.32mm, 0.25µm) coupled with mass spectrometer (Agilent 5975C). The injection was made in split mode (10:1) with an injection volume of 1µl. The injector temperature

was set to 240 °C. Helium was used as a carrier gas at a constant flow rate of 1 ml/min. The initial oven temperature was held at 50 °C for 5 min, then increased to 300 °C at a rate of 10 °C/min, and finally held at this temperature for 5 min (total time 35 min)

NMR analysis:

The synthesized compounds were dissolved in deuterated chloroform or dimethyl sulfoxide ($CDCl_3$ or $(CD_3)_2SO$). ^1H- and ^{13}C-NMR spectra were recorded at room temperature with a Bruker 400 MHz instrument, with chemical shifts reported in ppm relative to the residual deuterated solvent.

FTIR and UV spectroscopic analysis:

FTIR spectra were recorded in the Nicolet™ iS™ 10 FTIR Spectrometer (Thermo Scientific™). UV spectra were recorded by Tecan Infinite 200 PRO spectrophotometer.

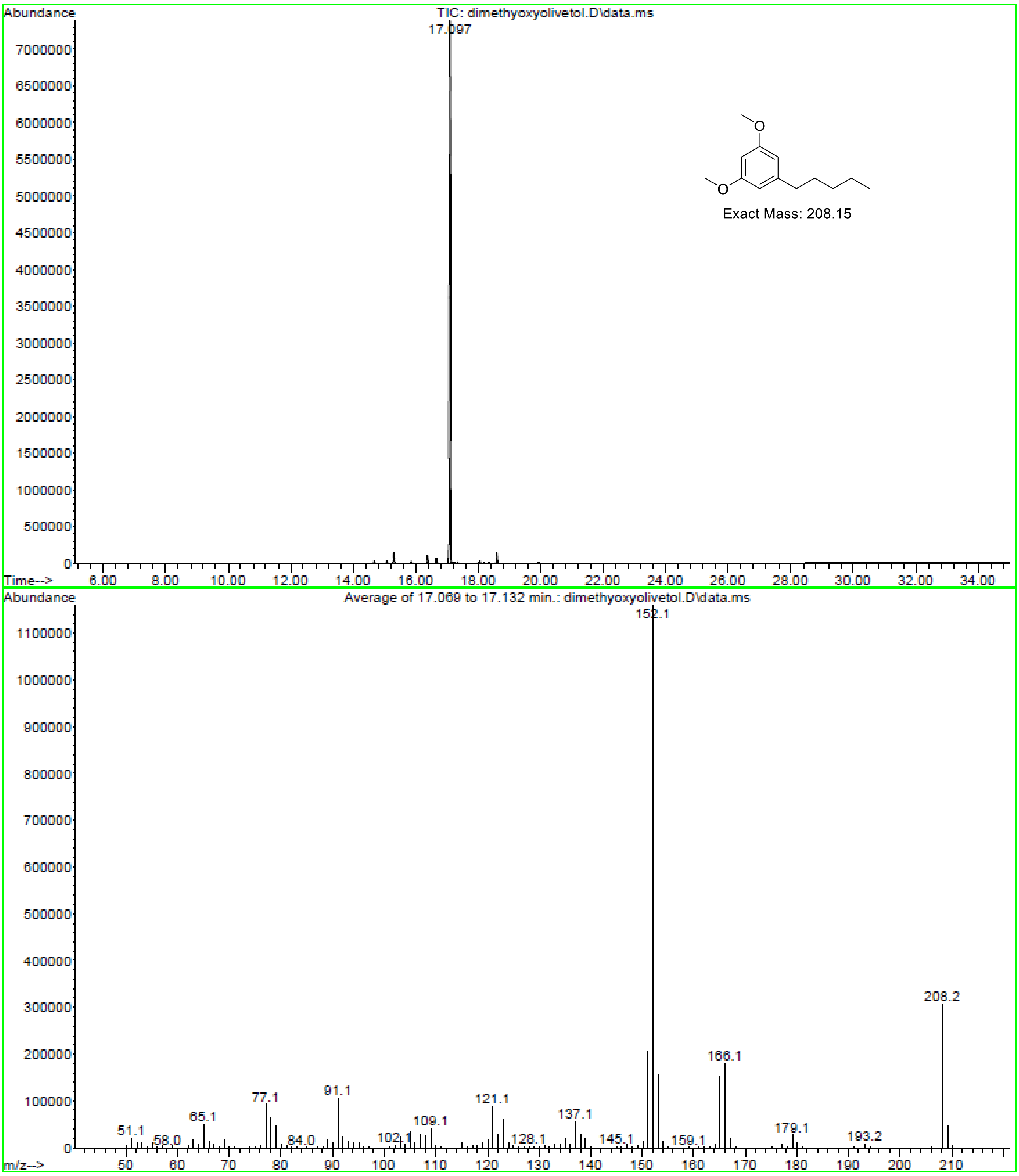

Figure S1. GC-MS chromatogram of 1,3-dimethoxy-5-pentylbenzene

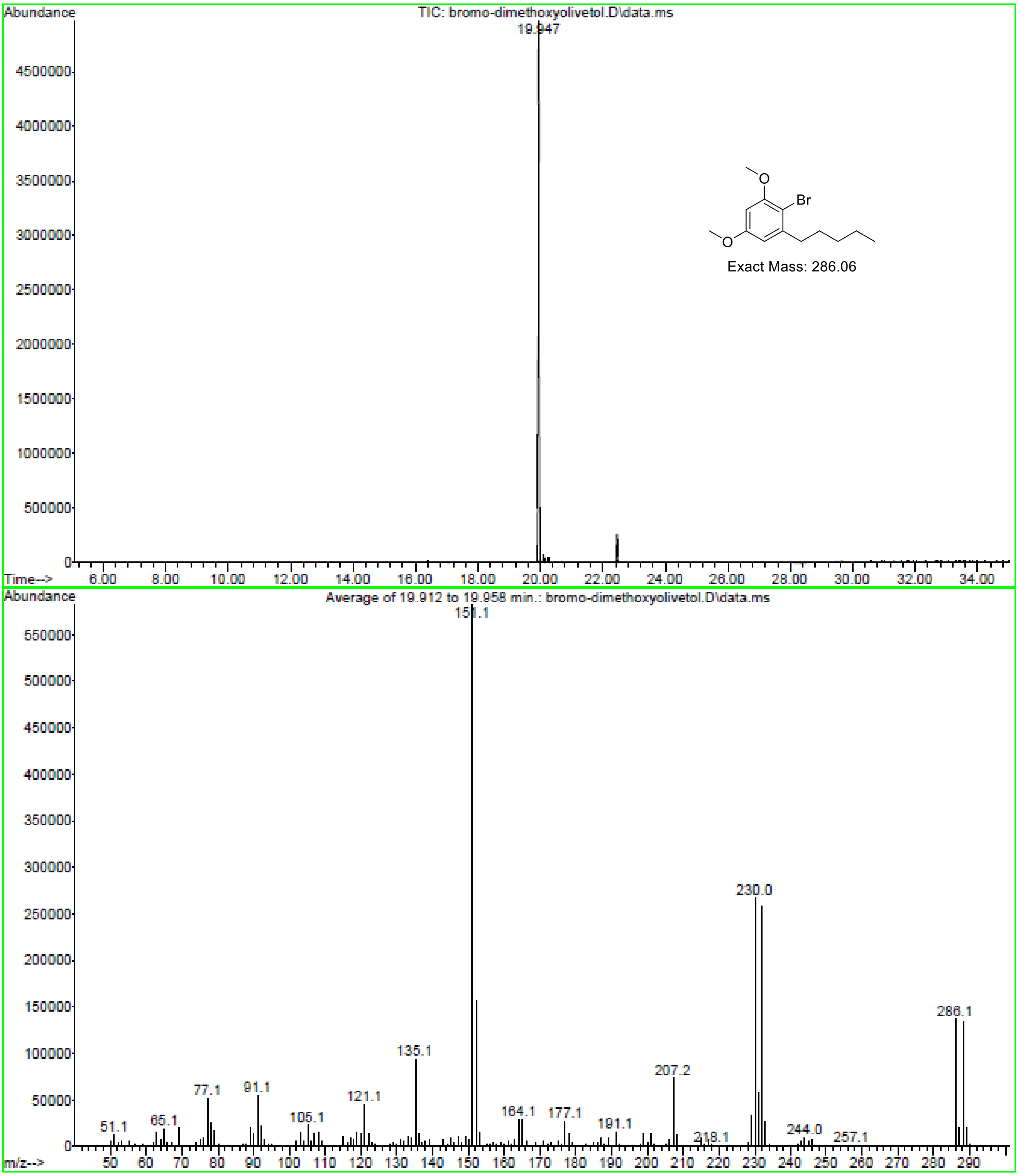

Figure S2. GC-MS chromatogram of 2-bromo-1,5-dimethoxy-3-pentylbenzene

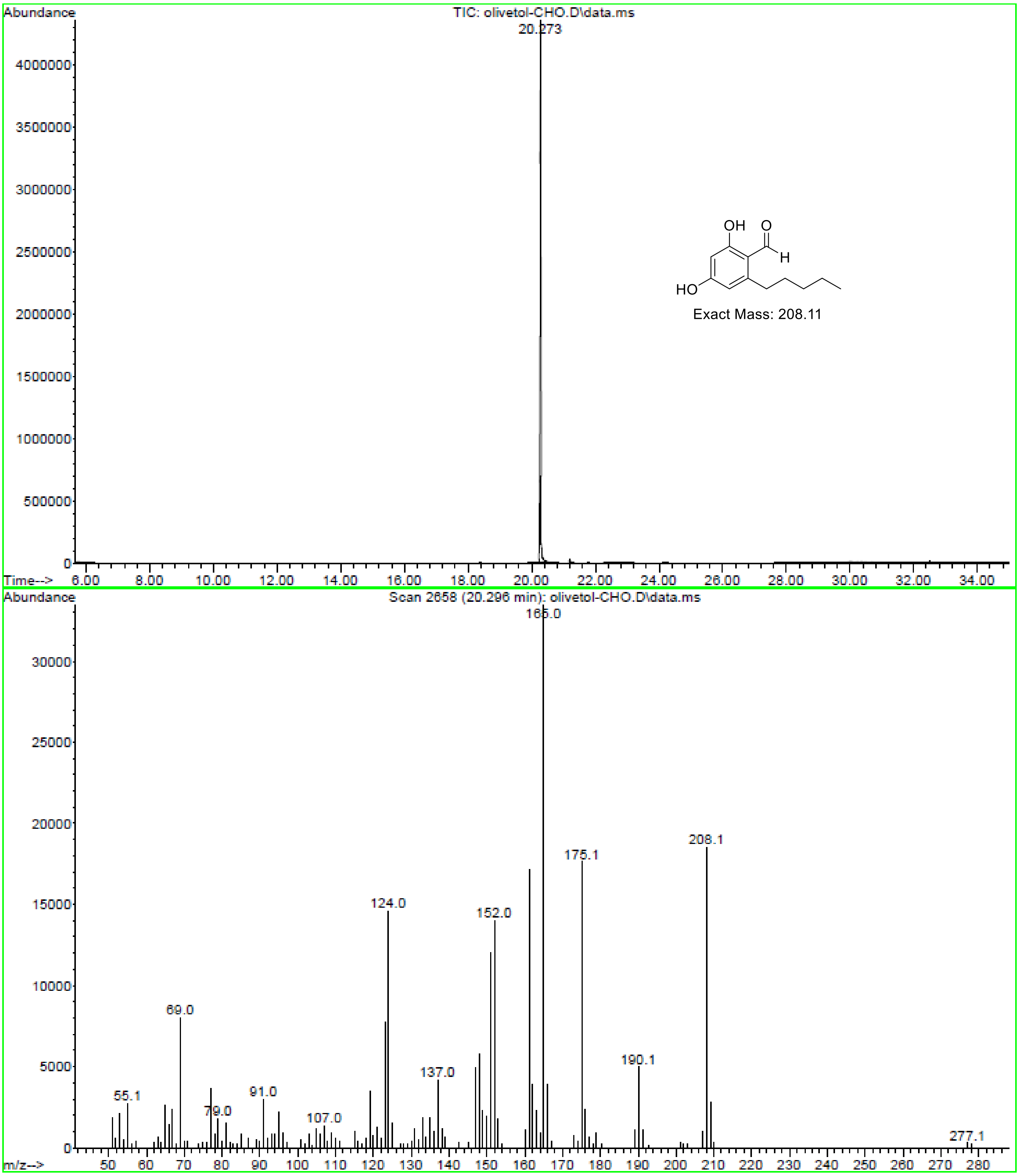

Figure S3. GC-MS chromatogram of 2,4-dihydroxy-6-pentylbenzaldehyde

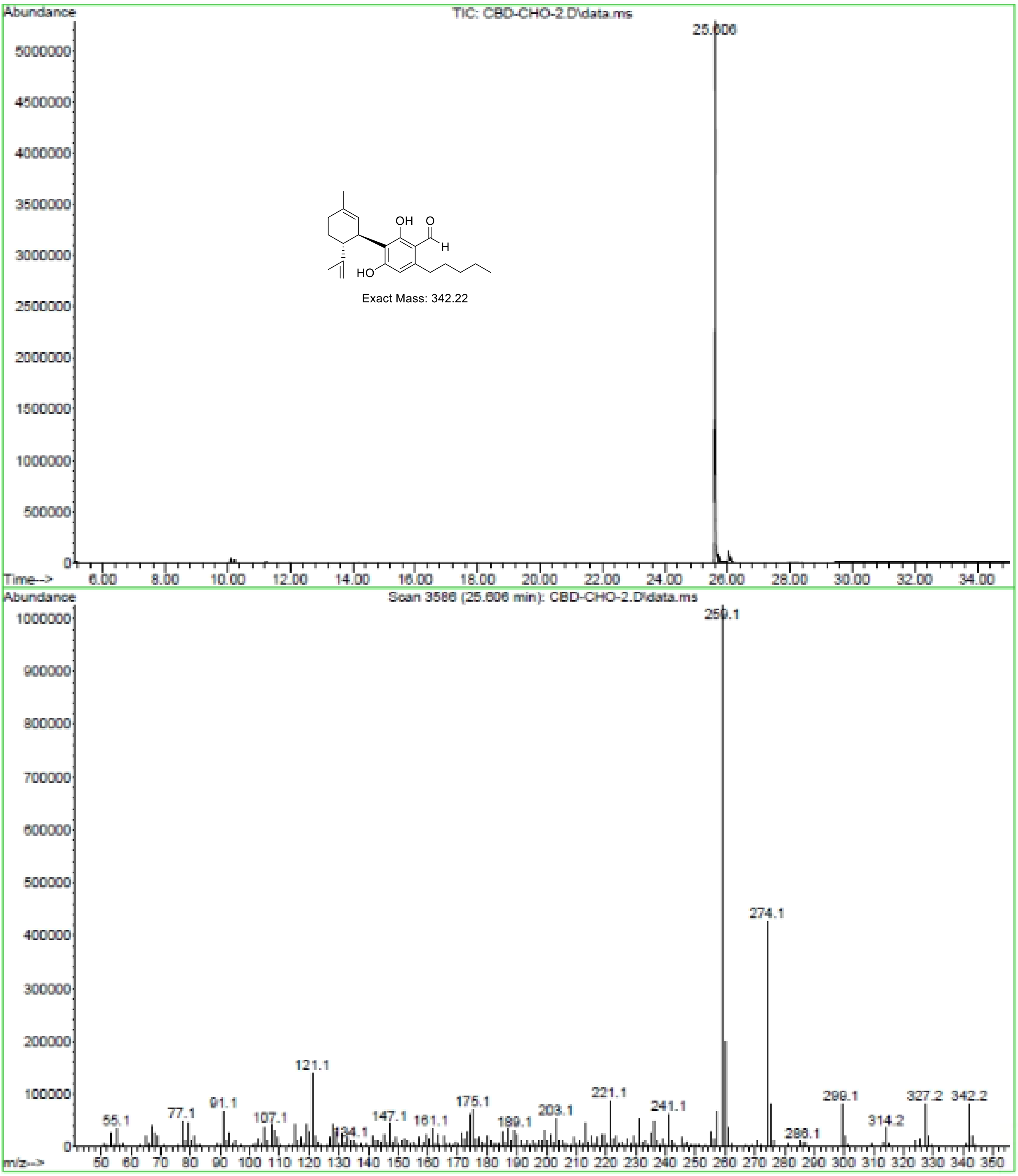

Figure S4. GC-MS chromatogram of 3-formyl cannabidiol

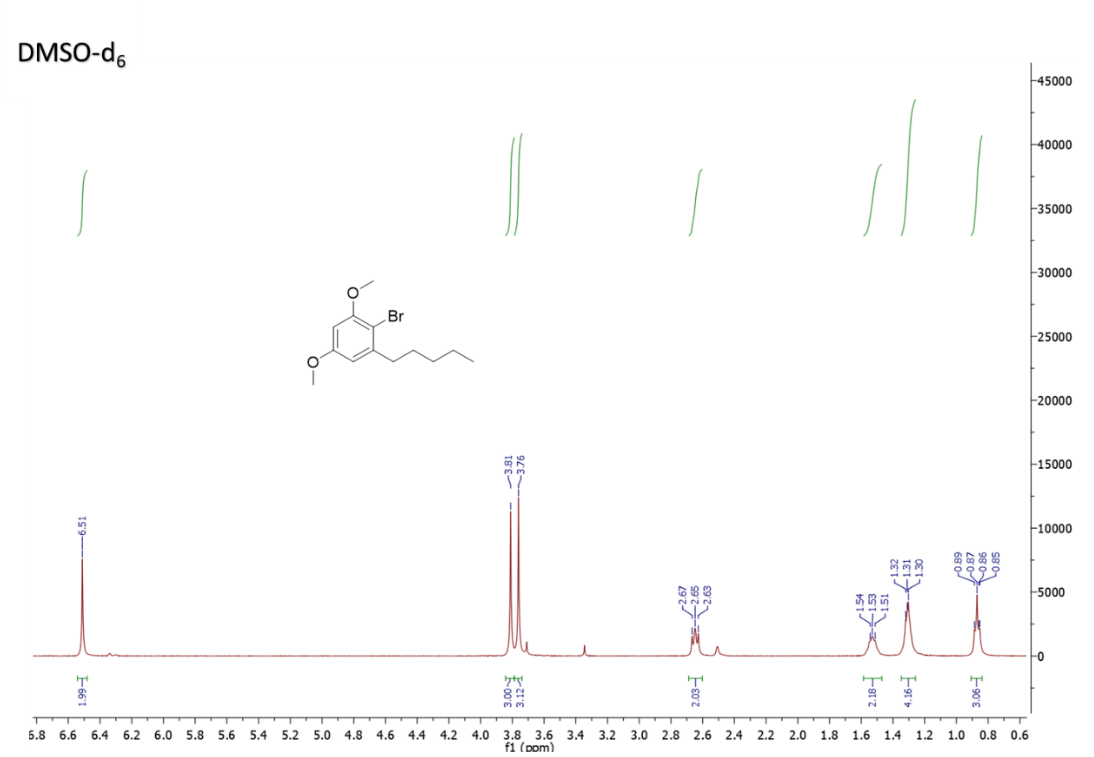

Figure S5. ^{1}H-NMR of 2-bromo-1,5-dimethoxy-3-pentylbenzene

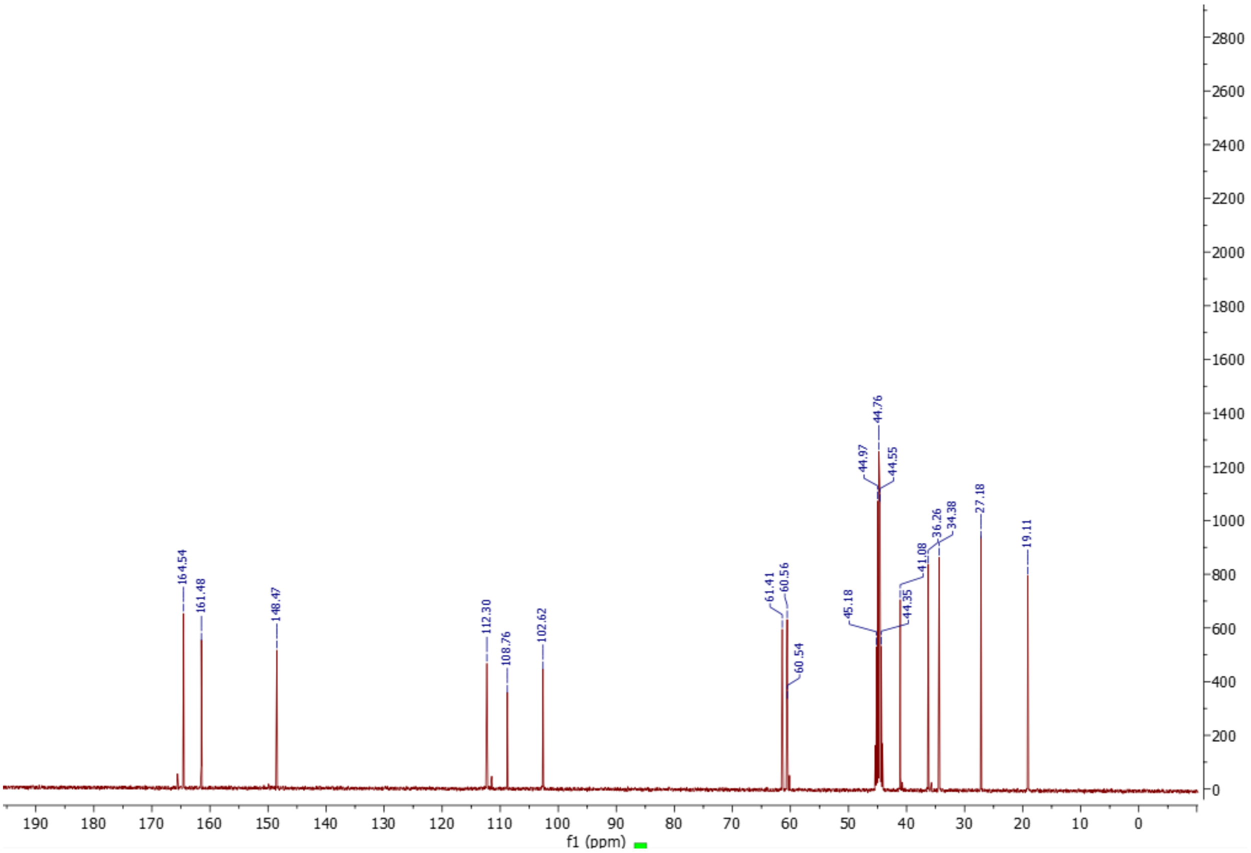

Figure S6. ^{13}C-NMR of 2-bromo-1,5-dimethoxy-3-pentylbenzene

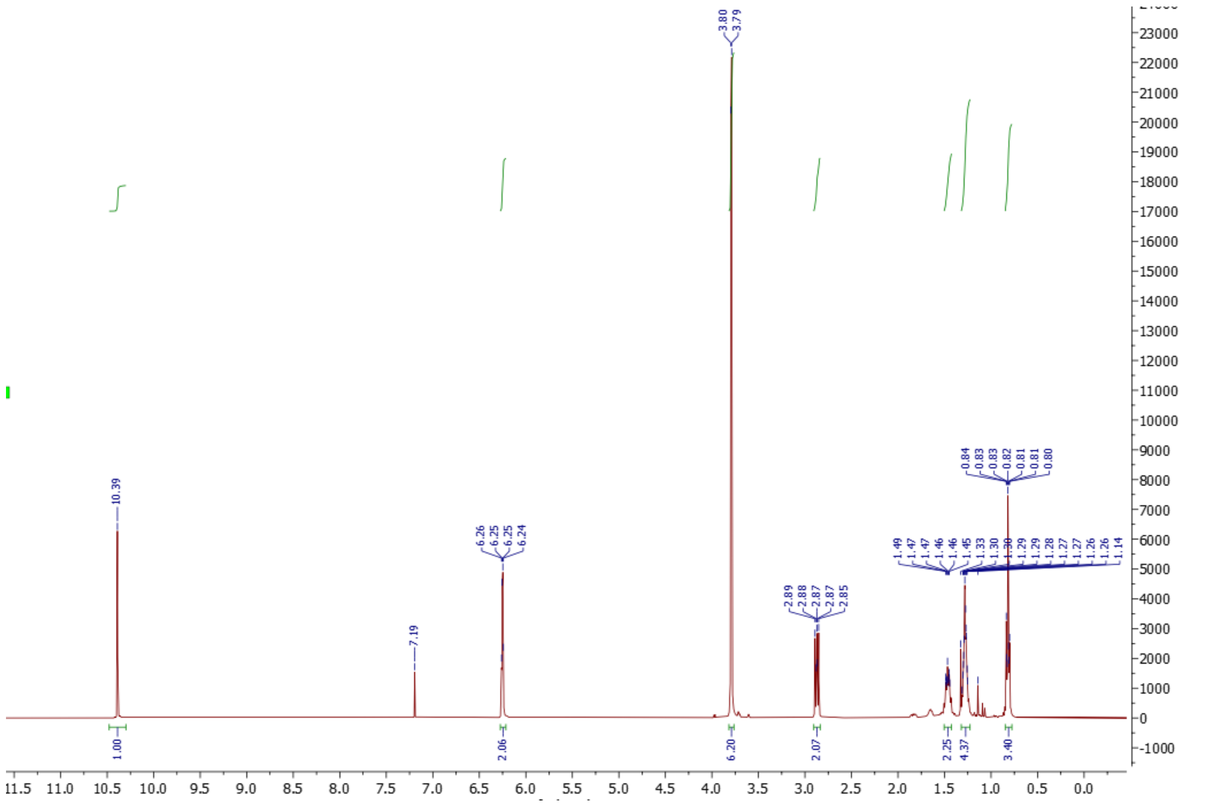

Figure S7. ^{1}H-NMR of 2,4-dimethoxy-6-pentylbenzaldehyde

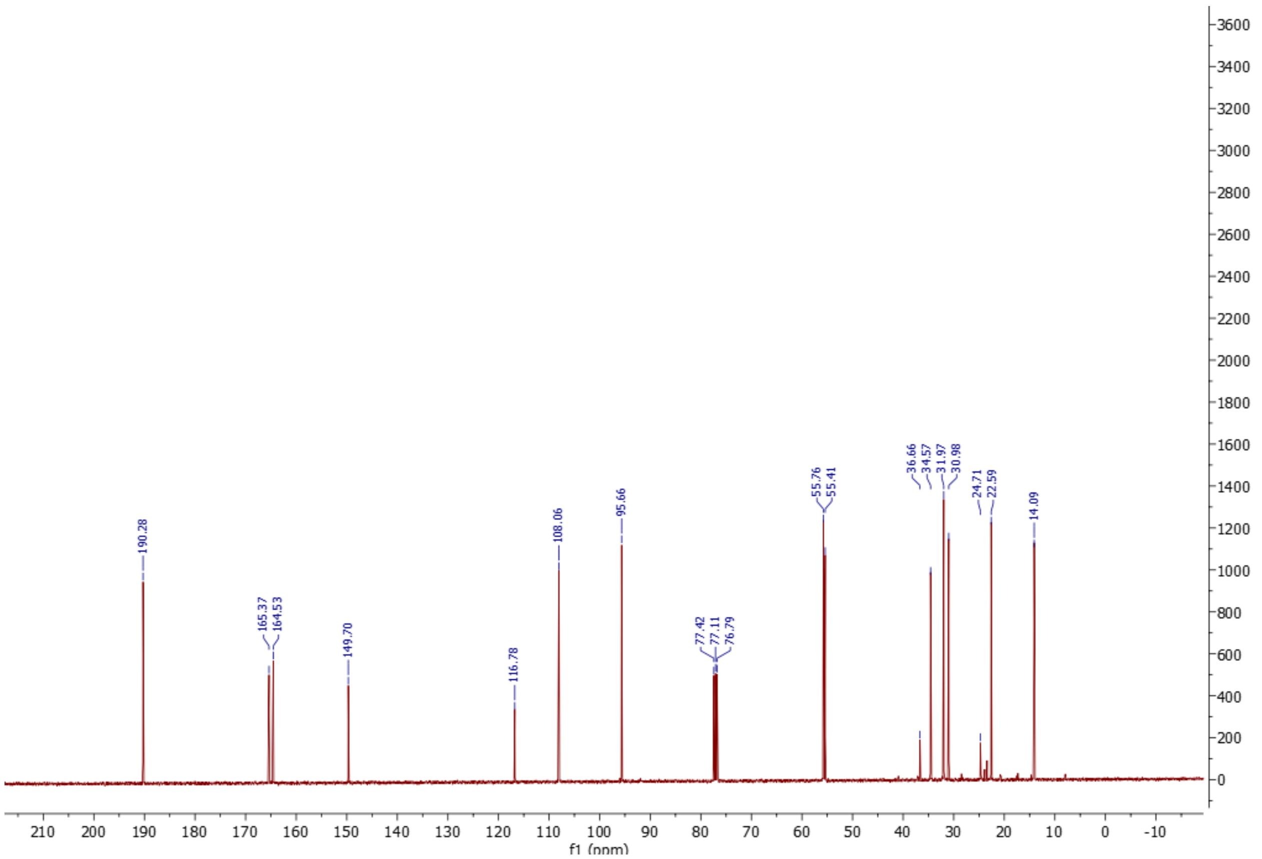

Figure S8. ^{13}C-NMR of 2,4-dimethoxy-6-pentylbenzaldehyde

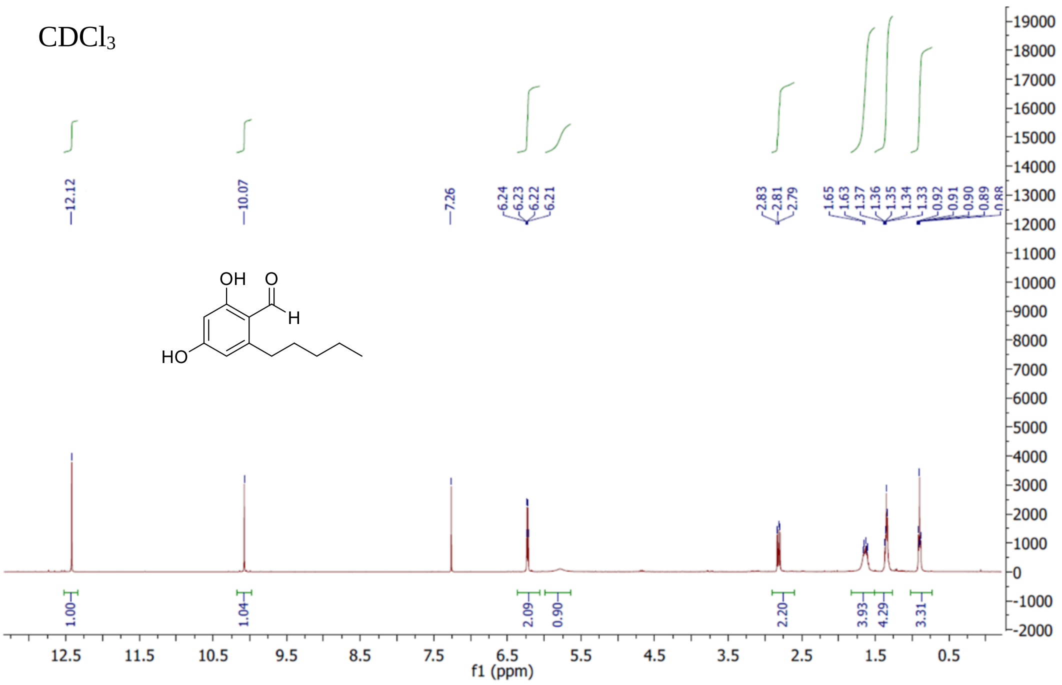

Figure S9. ^{1}H-NMR of 2,4-dihydroxy-6-pentylbenzaldehyde

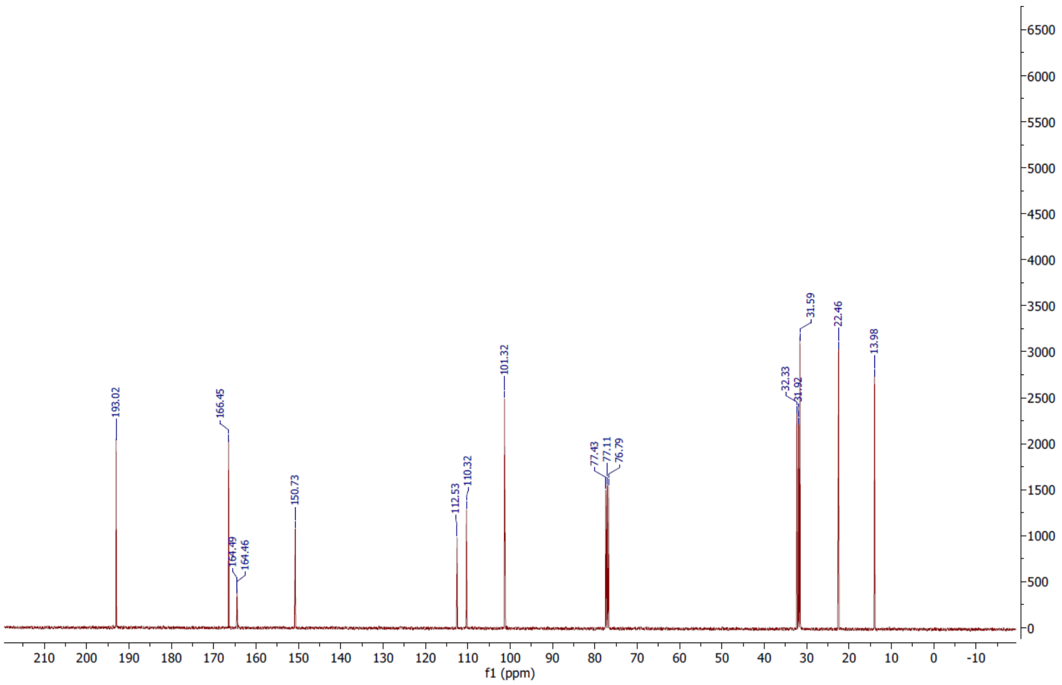

Figure S10. ^{13}C-NMR of 2,4-dihydroxy-6-pentylbenzaldehyde

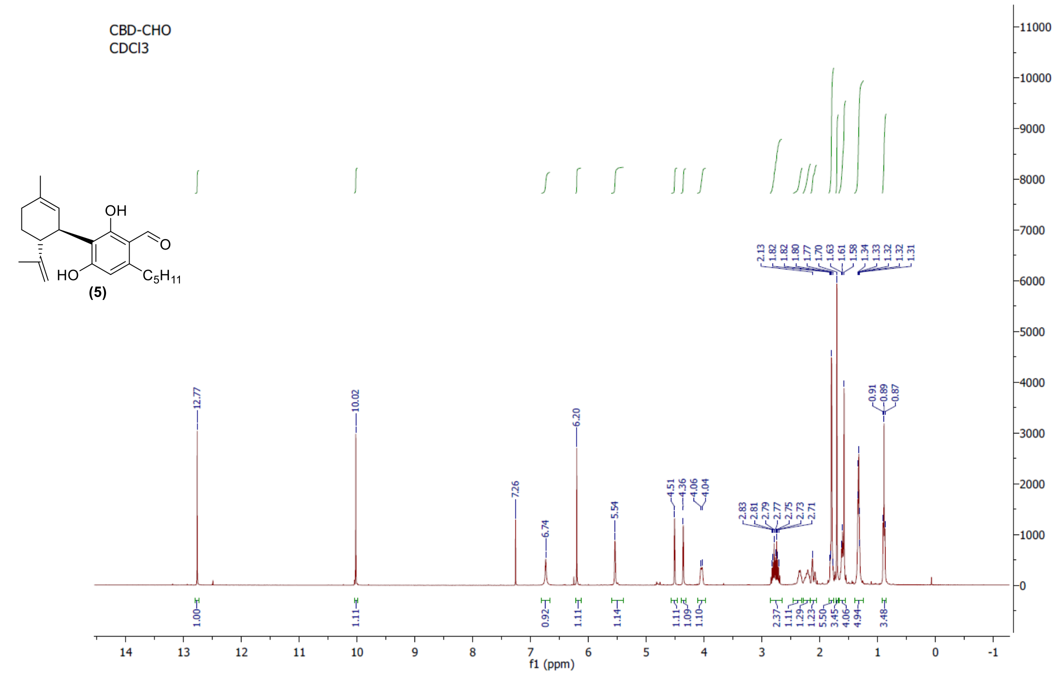

Figure S11. ^{1}H-NMR of 3-formyl cannabidiol

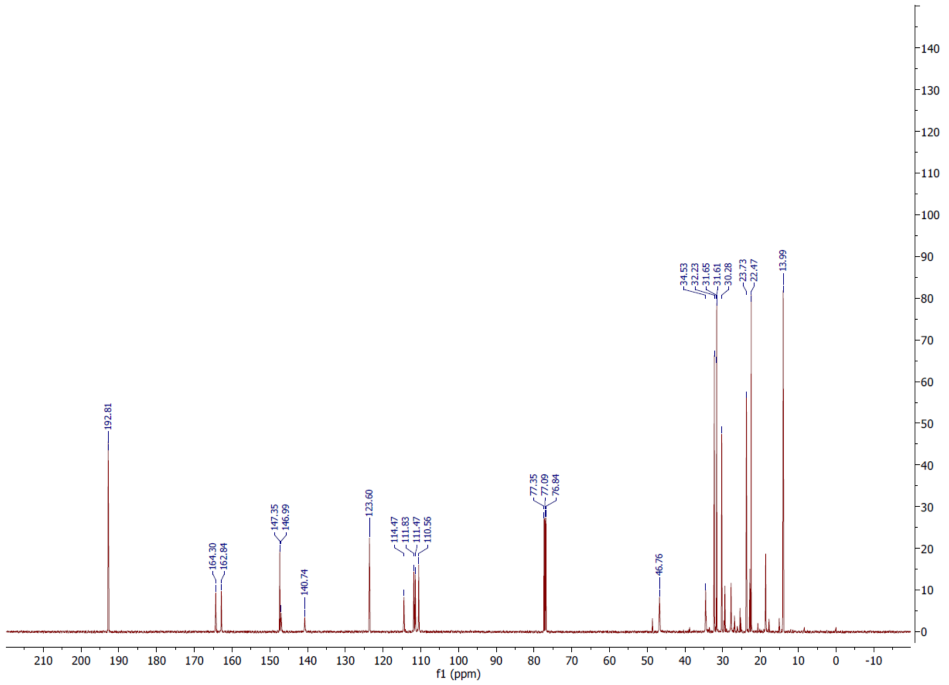

Figure S12. ^{13}C-NMR of 3-formyl cannabidiol

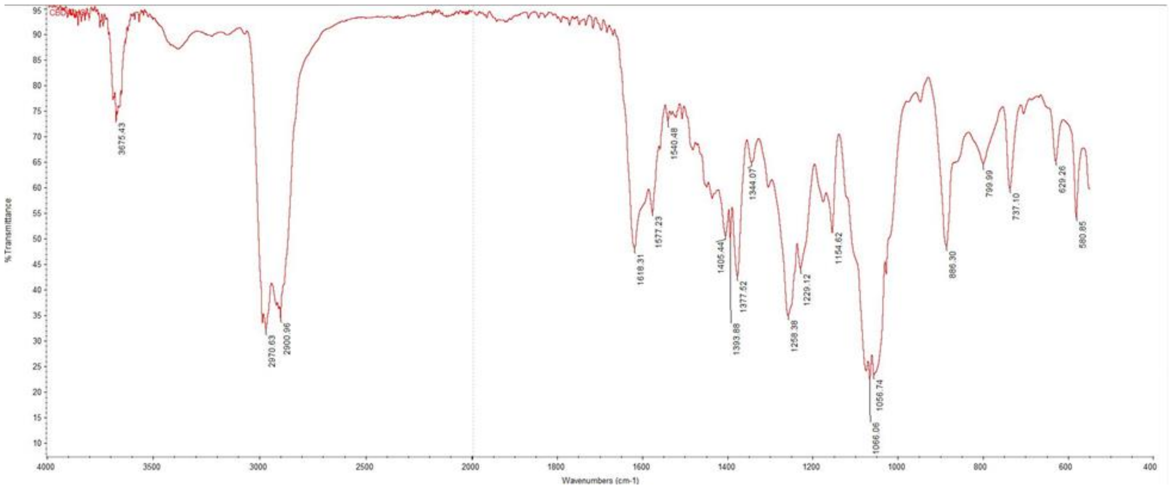

Figure S13. FTIR of 3-formyl cannabidiol

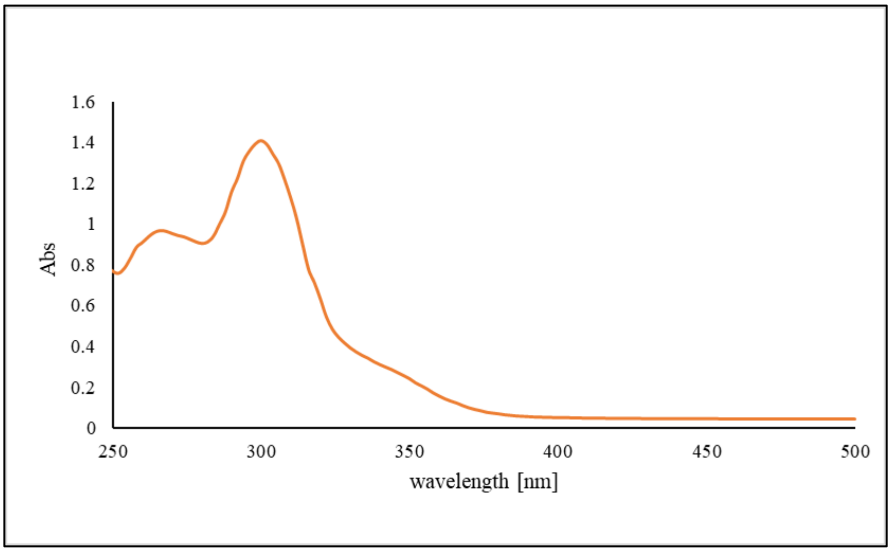

Figure S14. UV spectra of 3-formyl cannabidiol

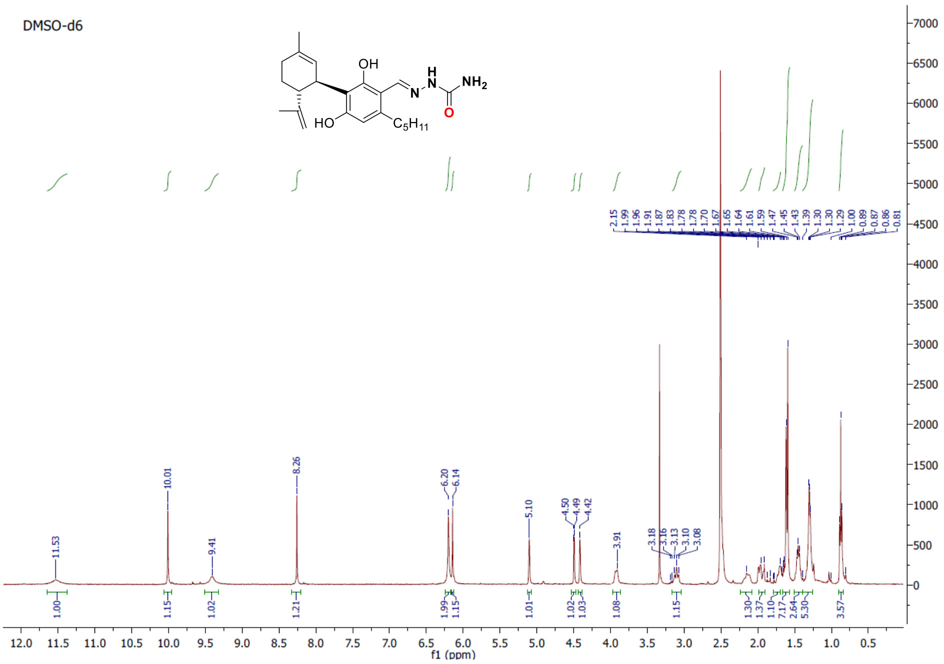

Figure S15. ^{1}H-NMR of 3-semicarbazone-CBD-aldehyde

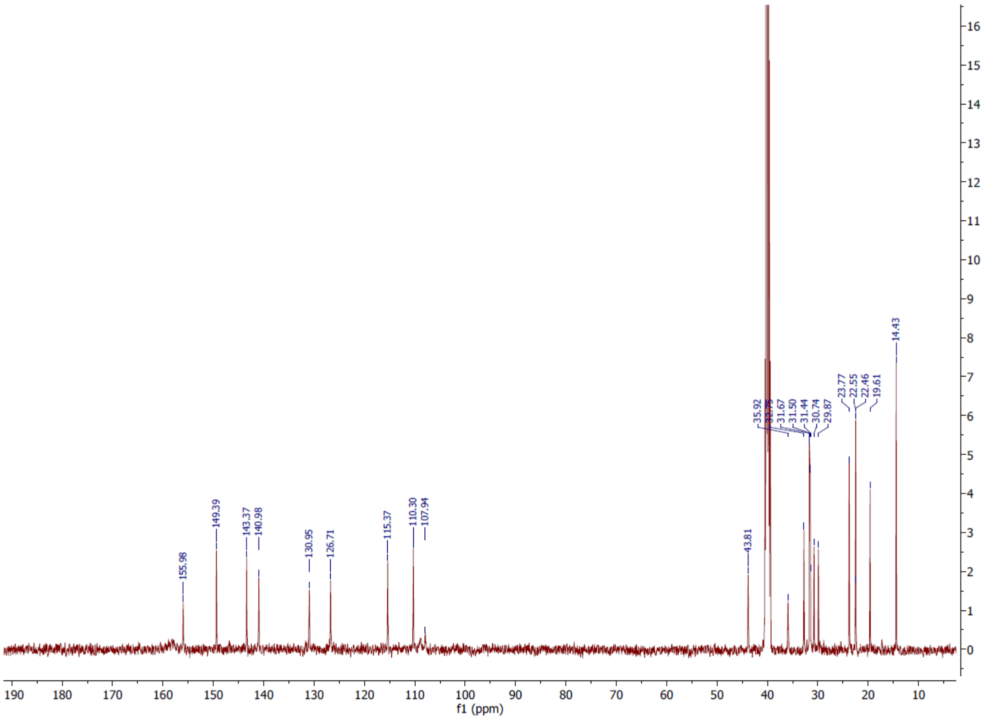

Figure S16. ^{13}C-NMR of 3-semicarbazone-CBD-aldehyde

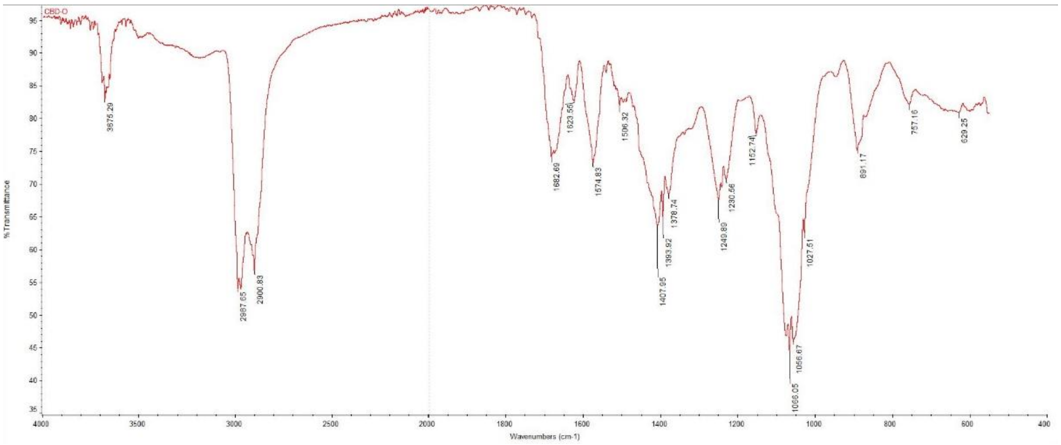

Figure S17. FTIR of 3-semicarbazone-CBD-aldehyde

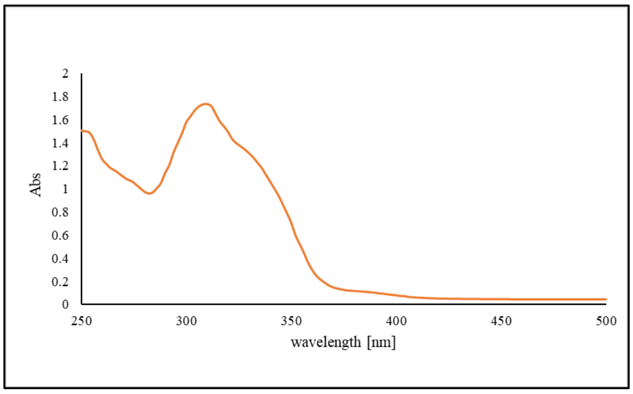

Figure S18. UV spectra of 3-semicarbazone-CBD-aldehyde

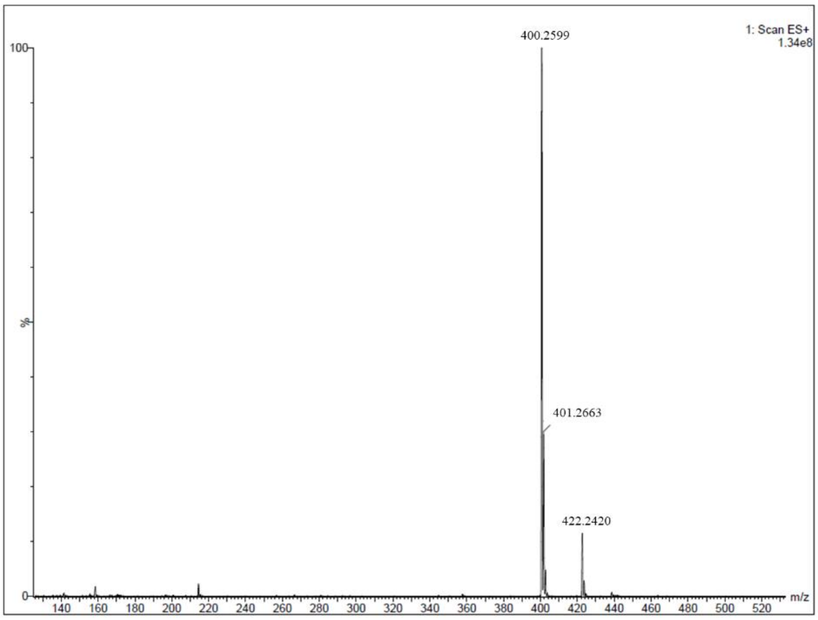

Figure S19. MS spectra of 3-semicarbazone-CBD-aldehyde

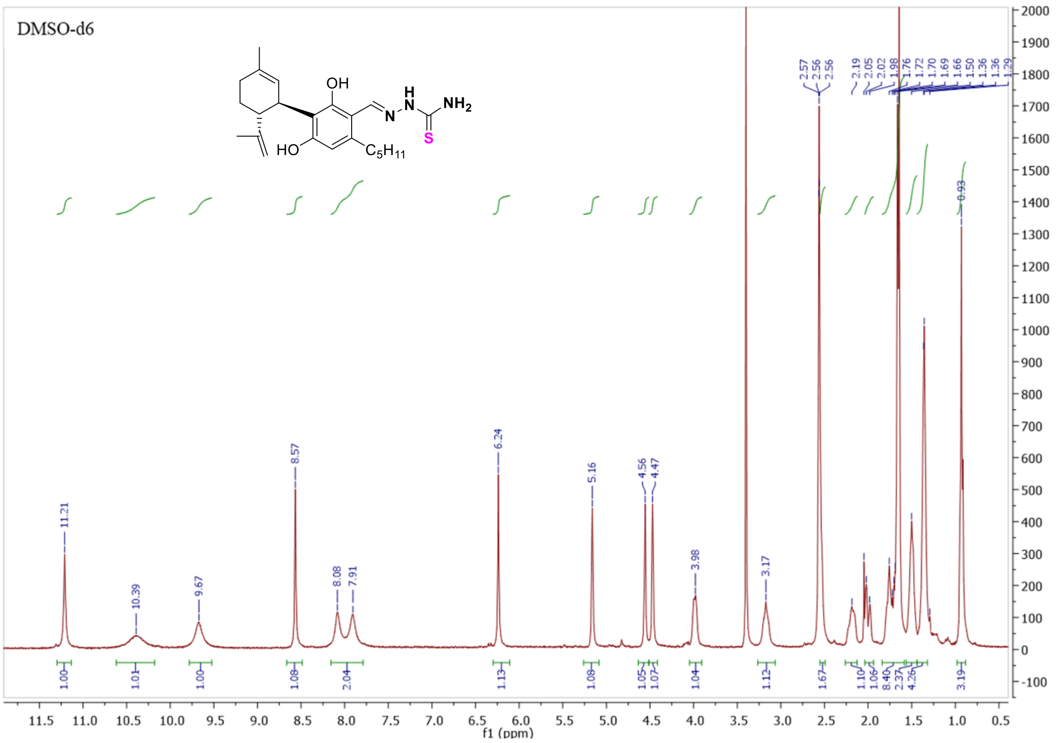

Figure S20. ^{1}H-NMR of 3-thiosemicarbazone-CBD-aldehyde

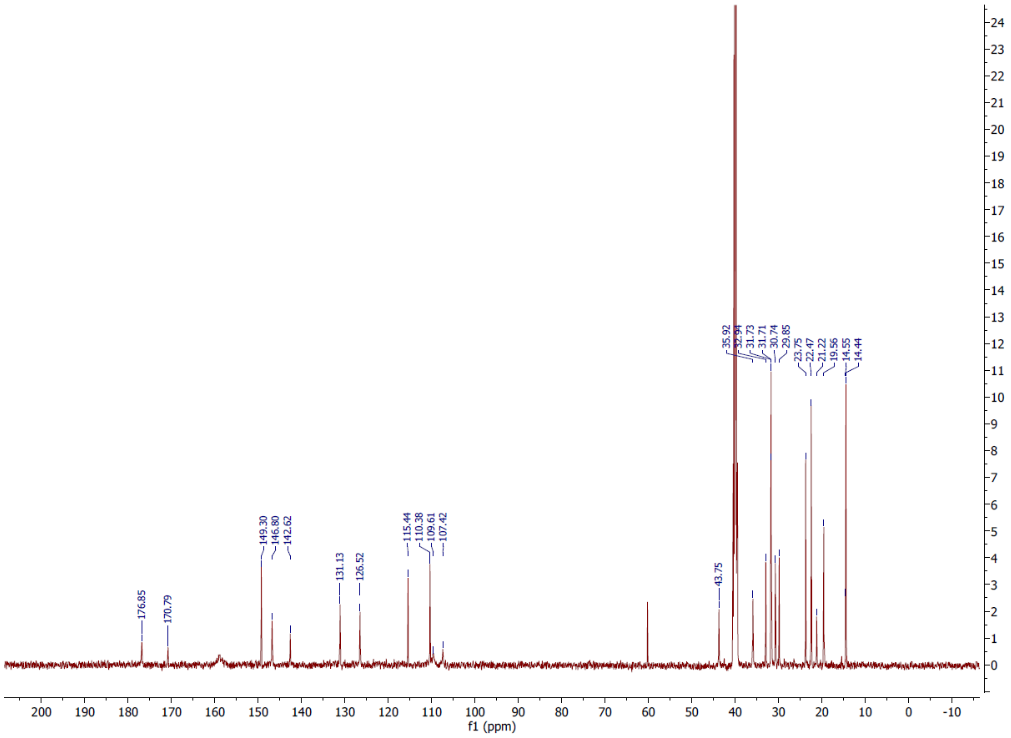

Figure S21. ^{13}C-NMR of 3-thiosemicarbazone-CBD-aldehyde

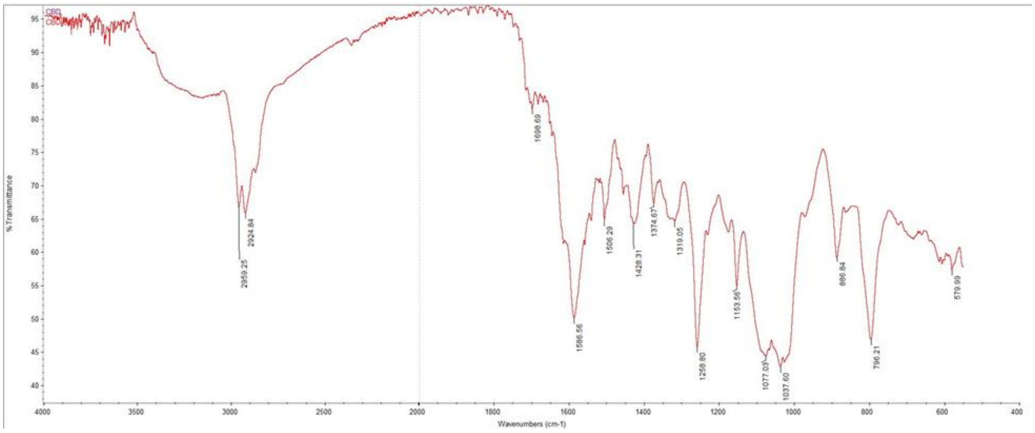

Figure S22. FTIR of 3-thiosemicarbazone-CBD-aldehyde

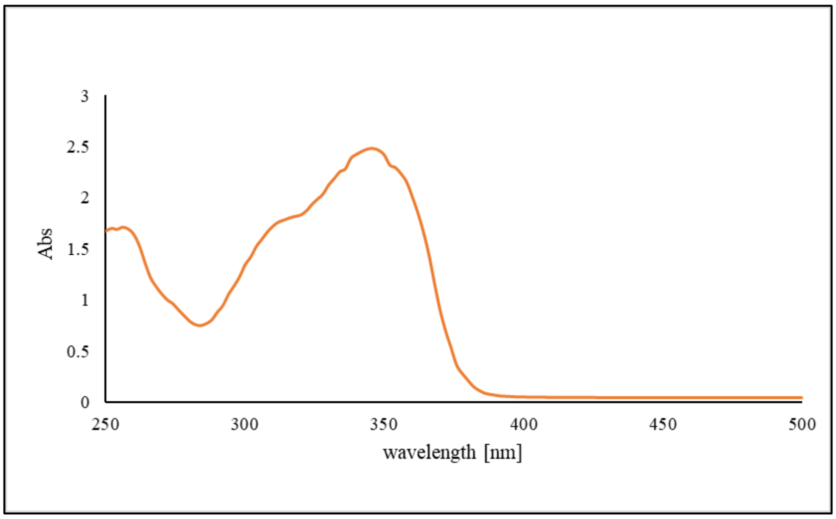

Figure S23. UV spectra of 3-thiosemicarbazone-CBD-aldehyde

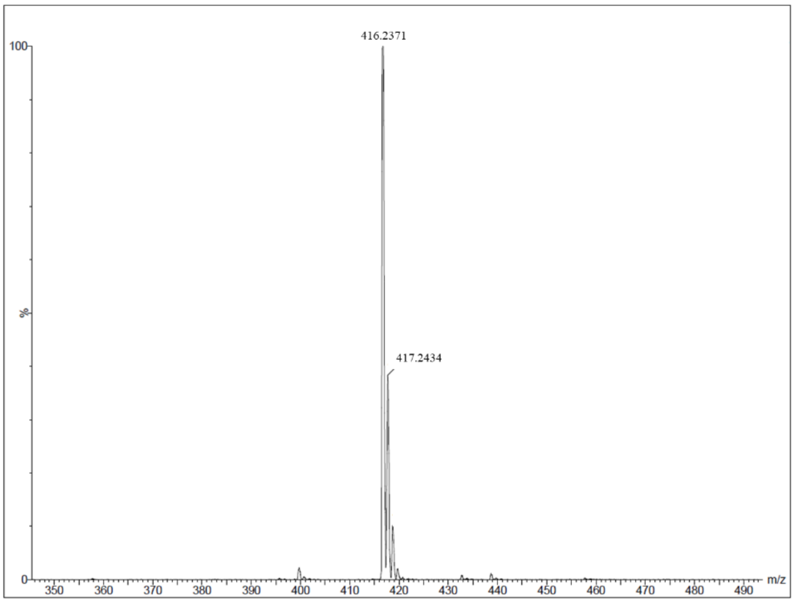

Figure S24. MS spectra of 3-thiosemicarbazone-CBD-aldehyde

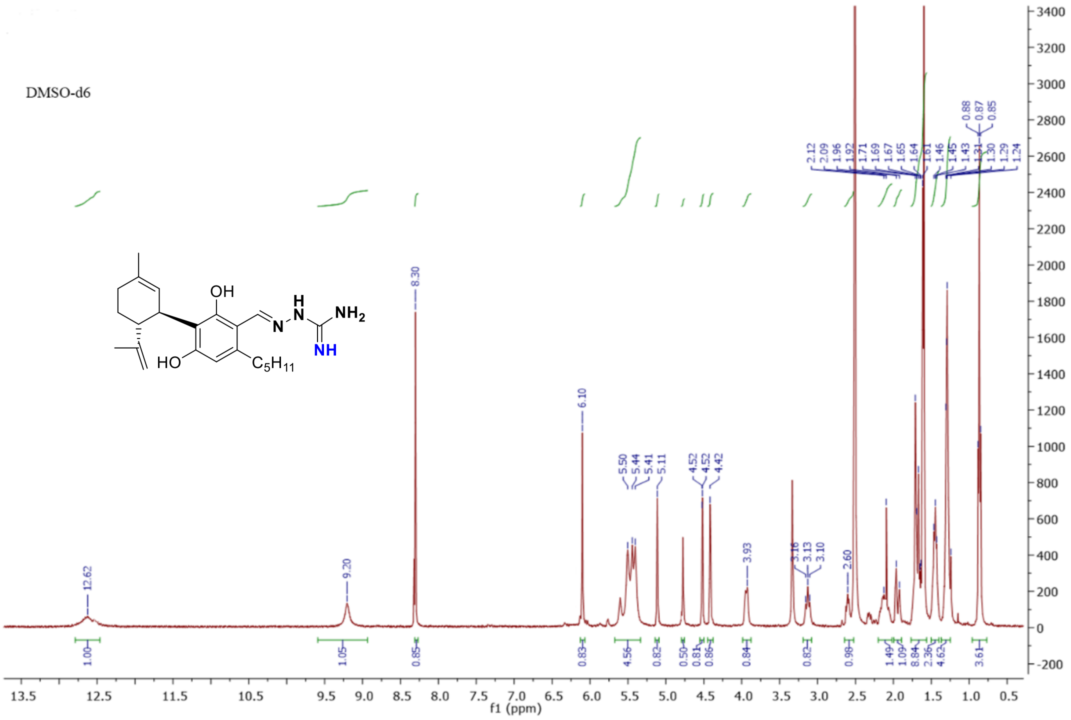

Figure S25. ^{1}H-NMR of 3-aminoguanylhydrazone-CBD-aldehyde

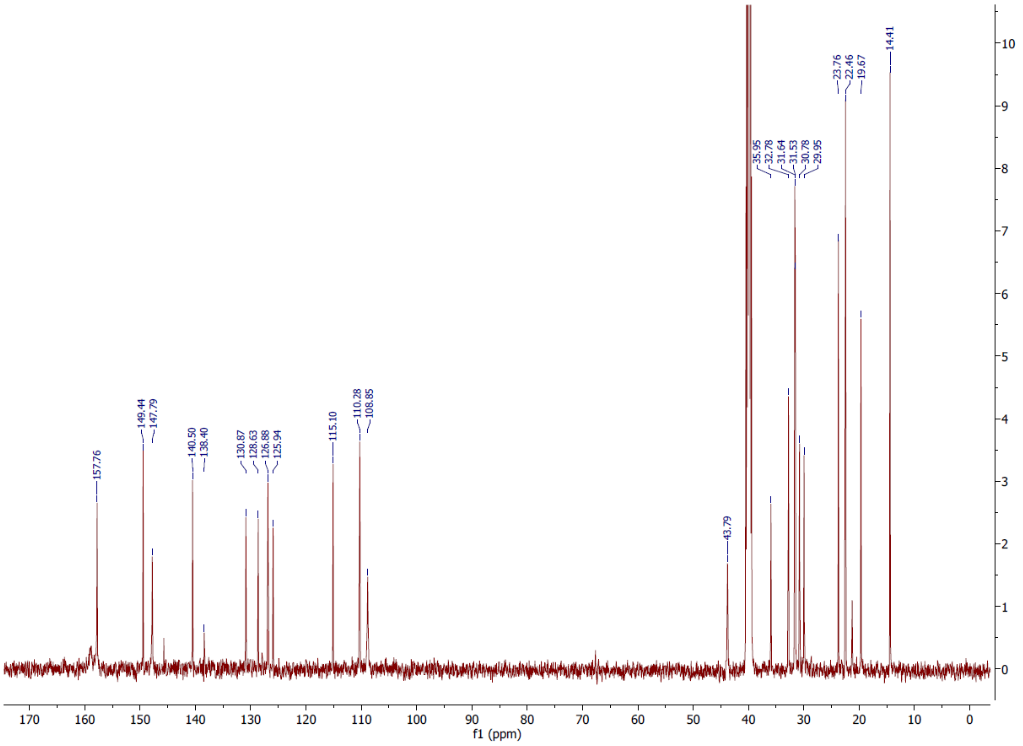

Figure S26. ^{13}C-NMR of 3-aminoguanylhydrazone-CBD-aldehyde

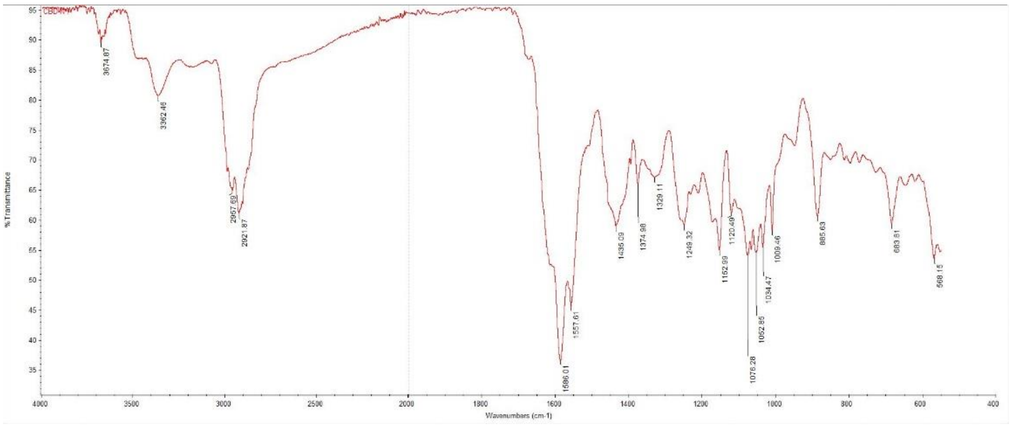

Figure S27. FTIR of 3-aminoguanylhydrazone-CBD-aldehyde

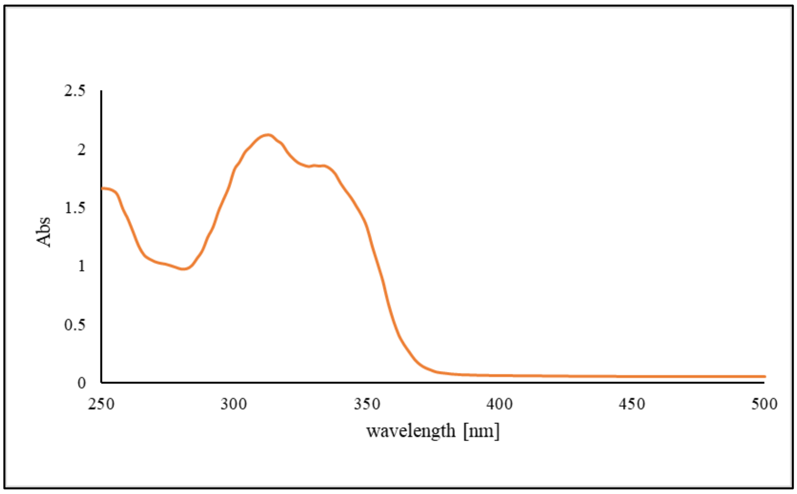

Figure S28. UV spectra of 3-aminoguanylhydrazone-CBD-aldehyde

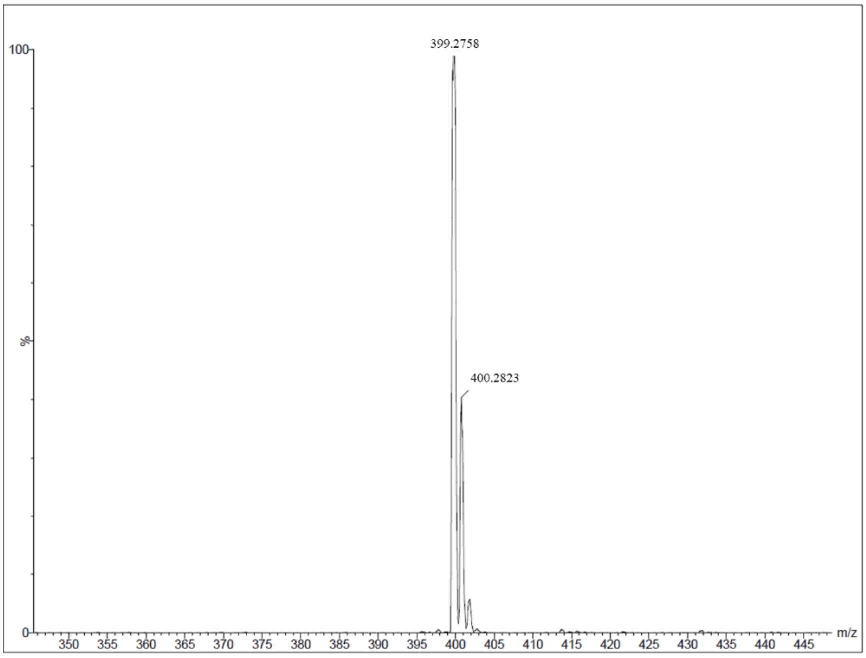

Figure S29. LCMS spectra of 3- aminoguanylhydrazone-CBD-aldehyde
